# Genome‐Wide Association Analysis Identifies *LILRB2* Gene for Pathological Myopia

**DOI:** 10.1002/advs.202308968

**Published:** 2024-08-29

**Authors:** Lingxi Jiang, Lulin Huang, Chao Dai, Rui Zheng, Masahiro Miyake, Yuki Mori, Shin‐ya Nakao, Kazuya Morino, Kenji Ymashiro, Yang‐Bao Miao, Qi Li, Weiming Ren, Zimeng Ye, Hongjing Li, Zhenglin Yang, Yi Shi

**Affiliations:** ^1^ Sichuan Provincial Key Laboratory for Human Disease Gene Study and the Center for Medical Genetics Department of Laboratory Medicine Sichuan Academy of Medical Sciences and Sichuan Provincial People's Hospital University of Electronic Science and Technology of China Chengdu Sichuan 610072 China; ^2^ Research Unit for Blindness Prevention of Chinese Academy of Medical Sciences (2019RU026) Sichuan Academy of Medical Sciences and Sichuan Provincial People's Hospital Chengdu Sichuan 610072 China; ^3^ Department of Ophthalmology and Visual Sciences Kyoto University Graduate School of Medicine Kyoto 606‐8501 Japan; ^4^ School of Medicine University of Sydney Camperdown NSW 2050 Australia; ^5^ Jinfeng Laboratory, Chongqing, China Chongqing 400000 China

**Keywords:** GWAS, LILRA3, LILRB2, lipid accumulation, pathological myopia

## Abstract

Pathological myopia (PM) is one of the leading causes of blindness, especially in Asia. To identify the genetic risk factors of PM, a two‐stage genome‐wide association study (GWAS) and replication analysis in East Asian populations is conducted. The analysis identified *LILRB2* in 19q13.42 as a new candidate locus for PM. The increased protein expression of LILRB2/Pirb (mouse orthologous protein) in PM patients and myopia mouse models is validated. It is further revealed that the increase in LILRB2/Pirb promoted fatty acid synthesis and lipid accumulation, leading to the destruction of choroidal function and the development of PM. This study revealed the association between *LILRB2* and PM, uncovering the molecular mechanism of lipid metabolism disorders leading to the pathogenesis of PM due to LILRB2 upregulation.

## Graphical abstract

Pathological myopia (PM) is a leading cause of blindness. To uncover the genetic basis of PM, an extensive two‐stage genome‐wide association study and replication analysis in East Asian populations is conducted. These findings reveal that elevated expression of LILRB2/Pirb disrupts lipid metabolism, impairing choroidal function, and promoting PM progression. These insights pave the way for innovative therapeutic strategies targeting PM.

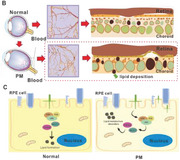

## 1 Introduction

Pathological myopia (PM) is one of the leading causes of blindness worldwide, particularly in Asia and the Middle East.^[^ ^1^ ^]^ A prominent characteristic of PM is the gradual elongation of the axial length (AL); this elongation persists over the course of the condition and can lead to various ocular complications when the AL exceeds 26 mm.^[^ ^2^ ^]^ Histopathological studies have identified several ocular complications associated with PM, such as tigroid fundus, lacquer cracks, geographic atrophy of the retinal pigment epithelium (RPE) and choroid,^[^ ^1^, ^3^ ^]^ as well as posterior staphyloma, choroidal neovascularization, myopic optic nerve head configuration, macular holes, and retinal detachments.^[^ ^2^, ^4^ ^]^ However, the pathogenic mechanisms of PM remain unclear. Recent research indicates that the choroid, located between the retina and the sclera, plays an important role in supplying oxygen to the sclera and is key in the development of PM and its complications.^[^ ^5^, ^6^ ^]^ Therefore, studying the choroid and its specific function in PM regulation is essential for understanding the pathogenesis of PM.

Both genetic and environmental factors play essential roles in the development of myopia, especially PM.^[^ ^7^ ^]^ Multiple family‐based whole‐genome linkage analyses have identified ≈27 loci associated with myopia, including but not limited to Xq28,^[^ ^8^ ^]^ 12q21‐23,^[^ ^9^ ^]^ 18p11.31,^[^ ^10^ ^]^ 17q21‐22,^[^ ^11^ ^]^ 2q37.1,^[^ ^12^ ^]^ 4q22‐q27,^[^ ^13^ ^]^ Xq23‐q25,^[^ ^14^ ^]^ 10q21.1,^[^ ^15^ ^]^ 5p15.33‐p15.2,^[^ ^16^ ^]^ and 7p15.^[^ ^17^ ^]^ However, only a few genes have been identified as either pathogenic or potentially pathogenic for PM. In 2009, a GWAS found a new genetic locus for PM at 11q24.1 represented by rs577948, which includes the genes *BLID* and *LOC399959* within a 200‐kb DNA region.^[^ ^18^ ^]^ In 2013, two loci (rs13382811 in *ZFHX1B* and rs6469937 in *SNTB1*) showed highly suggestive evidence of association with severe myopia.^[^ ^19^ ^]^ A recent GWAS has revealed that scleral *HIF‐1* α is a prominent regulatory candidate for genetic and environmental interactions in the pathogenesis of human myopia.^[^ ^20^ ^]^ To ascertain the genetic predisposing factors of PM, here, we conducted a two‐stage GWAS, which resulted in the identification of *LILRA3/LILRB2* in locus 19q13.42 as novel candidate genes for PM. Subsequent experimental investigations confirmed that LILRB2, which possesses homologous proteins (Pirb) in mice, modulates the core protein involved in choroidal lipid metabolism through the activation of the ERK–P38–JNK signaling pathway, thereby promoting the development of choroidal cavities and impairing vision. This study offers fresh insights and avenues for further exploration into the pathogenesis of PM.

## 2 Results

### 2.1 Characterization of the Patients with PM

A total of 2119 PM patients with an AL greater than 26.0 mm in both eyes were enrolled in this study. To maximize the detection power, the patients with an AL greater than 26.0 mm in both eyes were enrolled in the first stage of GWAS screening. Information of the cases and controls who passed quality‐control procedures of genotyping (see Experimental Section) is presented in **Table** **1**.

**Table 1:** Demographic and clinical characteristics of cases and controls.

Dataset	Total		Discovery stage (Sichuan)		Replication 1 (Shanghai)		Replication 2 (Guangzhou)		Replication 3 (Japan)	
	cases	controls	cases	controls	Cases	controls	cases	controls	cases	controls
Number of samples	2119	6033	806	2591	436	805	411	375	466	2262
Female	1235	3486	454	1429	236	359	225	149	320	1549
Male	884	2547	352	1162	200	446	186	226	146	713
Average age (years old)	45.12 ± 18.30	58.78 ± 18.05	30.89 ± 20.20	73.67 ± 25.82	37.73 ± 20.17	57.15 ± 18.45	43.2 ± 21.87	57 ± 18.44	56.89 ± 14.61	57.53 ± 13.78
Refractive errors (Diopters)										
OD^a)^	−12.08 ± 6.41		−11.62 ± 7.62		−12.62 ± 6.19		−12.41 ± 6.02		−11.88 ± 6.04	
OS^a)^	−11.87 ± 6.12		−12.79 ± 7.46		−12.92 ± 5.07		−10.27 ± 5.54		−11.70 ± 6.26	
Axial length [mm]										
OD^a)^	29.21 ± 8.382		28.06 ± 2.49		28.63 ± 3.45		30.77 ± 32.23		29.285 ± 1.87	
OS^a)^	29.05 ± 8.6		28.04 ± 2.34		28.68 ± 2.71		30.42 ± 30.04		29.06 ± 3.973	

^a)^ OD: Right eye; OS: Left eye.

### 2.2 Genome‐Wide Association Study and Replication Analysis

**Figure 1:**
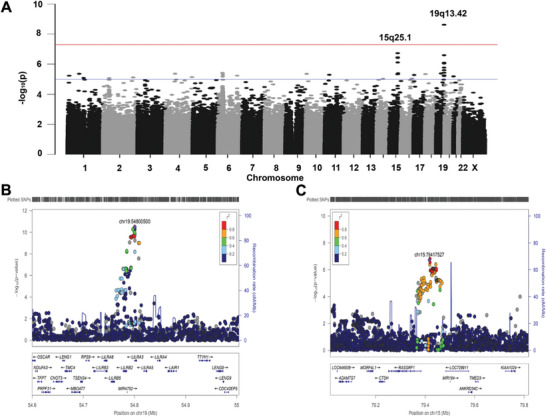
Genome‐wide association analysis (GWAS)‐associated regions from the discovery stage. A) Manhattan plots of the GWAS discovery stage for PM. Each plot shows −log10‐transformed *P* values for all SNPs. SNPs in locus 19q13.42 passed the genome‐wide significance (*P *< 5 × 10^−8^). The orange suggestive line is at 1 × 10^−5^, and the blue suggestive line is at 5 × 10^−8^. B) LocusZoom plots of regional association of *LILRA3/LILRB2* (19q13.42) in PM in the discovery stage. C) LocusZoom plots of the regional association of *RASGRF* on 15q25.1 in PM in the discovery stage.

For the discovery stage, 806 cases and 2591 controls and 764939 SNPs passed the quality‐control criteria (see details in Materials and Methods) for further statistical analysis. The Genomic Control (GC) method (Devlin and Roeder, 1999) revealed only a slight inflation of the test statistics (GC parameter l = 1.068). Locus 19q13.42 showed a significant association with PM by GWAS (with adjusted *P* values below 5 × 10^−8^) (**Figure** **1**A; Figure S1, Supporting Information). We chose three SNPs (rs7247538, rs13345069, and rs367070) located in 19q13.42 for replication (Figure 1B and **Table** **2**). Two reported myopia SNPs located in 15q25.1 (rs4778879 and rs939658) were also included in the replication stage as they nearly reached genome‐wide significance (Figure 1C and Table 2). Consequently, a total of five SNPs were selected for replication in the second stage. Other GWAs‐data results are shown in Supporting Information.

**Table 2:** Association results for the five loci identified in this study.

CHR	SNP^a)^/Chr^a)^./ Gene/Region	A1/A2	Stage	F_A	F_U	*P*	OR^a)^	N	*P*‐meta	P(R)‐met^a)^	OR‐met^a)^	OR(R)‐met^a)^	Q‐met^a)^	I
15	rs745030	G/A	Discovery	0.5464	0.4902	2.80×10^−5^	0.798	4	1.18 × 10^−12^	1.18 × 10^−12^	0.7837	0.7837	0.9268	0
	15q25.1		Replication 1 Shanghai	0.4448	0.5149	8.63 × 10^−4^	0.755							
	*RASGRF1*		Replication 2 Guangzhou	0.4404	0.4933	3.56 × 10^−2^	0.808							
	intron		Replication 3 Japan	0.4257	0.4898	2.23 × 10^−5^	0.772							
15	rs35602654	A/T	Discovery	0.3383	0.4063	1.88 × 10^−7^	0.747	4	4.76 × 10^−11^	4.76 × 10^−11^	0.7716	0.7716	0.8141	0
	15q25.1		Replication 1 Shanghai	0.3402	0.3980	4.63 × 10^−3^	0.780							
	*LINC00971*		Replication 2 Guangzhou	0.3516	0.3938	8.41 × 10^−2^	0.835							
	intron		Replication 3 Japan	0.3398	0.3961	1.29 × 10^−2^	0.785							
19	rs7247538	T/C	Discovery	0.0401	0.0668	2.58 × 10^−5^	0.583	4	2.98 × 10^−12^	1.42 × 10^−9^	0.6282	0.6447	0.1953	36.13
	19q13.42		Replication 1 Shanghai	0.0280	0.0396	2.10 × 10^−2^	0.698							
	*LILRB2*		Replication 2 Guangzhou	0.0549	0.0689	03.35 × 10^−2^	0.784							
	p.His300Tyr		Replication 3 Japan	0.0008	0.0014	0.708	0.592							
19	rs13345069	A/C	Discovery	0.0564	0.0948	2.55 × 10^−7^	0.570	4	2.47 × 10^−12^	2.47 × 10^−12^	0.5836	0.5836	0.9263	0
	19q13.42		Replication 1 Shanghai	0.0632	0.1039	7.23 × 10^−4^	0.582							
	*LILRB2*		Replication 2 Guangzhou	0.0632	0.1041	7.23 × 10^−4^	0.582							
	Utr‐5		Replication 3 Japan	0.0188	0.0260	0.2835	0.715							
19	rs367070	A/G	Discovery	0.2682	0.3518	3.19 × 10^−11^	0.675	4	2.63 × 10^−22^	2.63 × 10^−22^	0.6819	0.6819	0.4265	0
	19q13.42		Replication 1 Shanghai	0.2989	0.4086	6.66 × 10^−8^	0.617							
	*LILRA3*		Replication 2 Guangzhou	0.4350	0.5285	2.33 × 10^−4^	0.687							
	intron		Replication 3 Japan	0.1548	0.1950	9.23 × 10^−4^	0.756							

^a)^ Abbreviations: SNP, single‐nucleotide polymorphism; CHR, chromosome; OR, odds ratio.

Of the reported SNPs associated with myopia in the GWAS Catalog (https://www.ebi.ac.uk/gwas/), a total of 2234 SNPs in linkage disequilibrium (LD) with 83 of the nine reported GWAS analyses of PM or myopia (Tables S2 and S3, Supporting Information) were included in the discovery study (Table S4, Supporting Information): including *ZEB2*,^[^ ^19^, ^36^ ^]^ *SNTB1* and *GJD2*.^[^ ^37^ ^]^ The SNPs with association *P* values in the reported LDs are listed in Tables S2–S4 (Supporting Information). Among them, the SNPs in *ZEB2* showed the strongest association (Table S4, Supporting Information).

For the replication analysis, 436 PM and 805 control subjects from Shanghai (replication 1), 411 PM and 375 control subjects from Guangzhou (replication 2), and 466 PM and 2276 control subjects from Japan (replication 3) were genotyped by TaqMan SNP assay. The genotyping success rates of the five SNP markers were 100%. The meta‐analysis results for the discovery and replications revealed that two loci (15q25.1 and 19q13.42) were significantly associated (*P*_meta_ < 5 × 10^−8^) with PMs (Table 2).

Five SNPs located at 15q25.1 and 19q13.42 reached the genome‐wide significance (*P *< 5 × 10^−8^, Table 2). Among them, rs367070 located in the intron of *LILRA3* (19q13.42) showed the strongest association (*P* = 2.63 × 10^−22^; odds ratio [OR]_Ｇ_ = 0.68) in the meta‐analysis (Table 2). In the same LD, rs7247538 (p. His300Tyr of *LILRB2*; *P* = 3.13 × 10^−10^; OR_T_ = 0.70) and rs13345069 (UTR‐5′ of *LILRB2*; *P* = 2.47 × 10^−12^; OR_A_ = 0.68) were also significantly associated with PM. In addition, rs745030 (in the intron of *RASGRF1*, 15q25.1), which was located in the same LD of the reported SNP rs4778879 for refractive error,^[^ ^38^ ^]^ showed an association with PM (*P* = 1.18 × 10^−12^; OR_G_ = 0.78). Another SNP rs35602654 (in the intron of *LINC00971*), which was in the same LD of the reported SNP rs939658 for refractive error,^[^ ^39^ ^]^ showed an association with PM (*P* = 4.76 × 10^−11^; OR_A_ = 0.77). Overall, the alternate bases of these five target SNPs appear to be protective against PM.

All of the five target candidate SNPs were predicted to alter the regulatory motif of transcription factors by the HaploReg version 4.1 database, suggesting their potential role in the regulation of gene expression. Additionally, the RegulomeDB database indicated that *RASGRF1*‐rs745030, *LINC00971*‐rs35602654, *LILRB2*‐rs7247538, and *LILRB2*‐rs13345069 showed strong evidence of regulatory function, with a RegulomeDB score of 2b, 3a, and 5, respectively, likely affecting transcription‐factor binding. However, there was no significant evidence of transcription‐factor binding at the other three SNPs (Table S3, Supporting Information). The GTEx Portal database showed that the three leading SNPs (*RASGRF1*‐rs745030, *LINC00971*‐rs35602654, and *LILRA3*‐rs367070) significantly affected gene expression, i.e., both rs745030 and rs35602654 affected the expression of *RASGRF1*, and rs367070 affected the expression of LILRB2 in the whole blood (Figure S2, Supporting Information). Indeed, according to the eQTL and sQTL data of GTEX, the change in the rs367070‐*LILRA3* locus affected the expression of most leukocyte immunoglobulin‐like receptor family members (e.g., *LILRB1*, *LILRP2*, *LILRA4*) but did not significantly affect the expression of *LILRA3*. Both results of eQTL and sQTL suggested that the rs367070‐*LILRA3* locus might have a more significant effect on the function of the *LILRB2* gene than that of *LILRA3* (Figure S2, Supporting Information).

### 2.3 Evaluation of the 19q13.42 Locus

Human chromosome 19q13.4 contains important immune‐cell receptor genes,^[^ ^40^ ^]^ such as killer cell inhibitory receptors (KIRs), leukocyte immunoglobulin (Ig)‐like receptors (LILRs), leukocyte‐associated Ig‐like receptors, and the Fca receptor (FcaR). The SNP rs367070 located at chromosome 19q13.42 showed a *P* = 2.63 × 10^−22^ by meta‐analysis with an OR of 0.68 for the protective allele A (*P* = 3.19 × 10^−11^ in the discovery stage; *P* = 6.66 × 10^−8^, *P* = 2.33 × 10^−4^, and *P* = 9.23 × 10^−4^ in each replication) (Table 2). Using the results of the first stage, an LD block that extending a 200‐kb region upstream and downstream of rs367070 was generated (Figure 1B). As shown in Figure 1B, two genes (*LILRA3* and *LILRB2*) were located in a 200‐kb region containing rs367070 and rs13345069 with an association signal.

In addition, according to the dbGap database, there were significant differences in the aggregated allele frequencies of the above two target SNPs in different populations. Among them, the frequency values of the risky rs13345059C‐*LILRB2* and rs367070‐G allele have evolved to the highest in East Asian populations with high myopia (Table S4, Supporting Information). The frequency of the rs13345059C allele in the Asian population was almost double the global average of this allele (rs13345059C allele = 0.843 in Asian populations; rs13345059C allele = 0.494 in global populations). The frequency of the rs367070G allele in Asian populations was more than twice the global average (rs367070G allele = 0.481 in Asian populations; rs367070G allele = 0.19157 in global populations). Such results explained the large population differences between the two target SNPs in the population.

### 2.4 Generalized Gene‐Set Analysis of GWAS Data by MAGMA

Gene‐set analysis of discovery GWAS results of PM versus control cases showed 31 enriched GO terms with *P* < 10^−5^ (significant terms after Bonferroni correction, Table S5, Supporting Information). Among them, the major histocompatibility complex (MHC) protein family (MHC complex, *P* = 6.5025 × 10^−7^; MHC‐class‐I protein complex, *P* = 1.5069 × 10^−5^; MHC‐class‐II protein complex, *P* = 2.8964 × 10^−5^) was the most enriched, suggesting a relationship between the ability of the immune system to respond to pathogens and PM.

### 2.5 The Expression of *LILRB2* is Increased in PM

**Figure 2:**
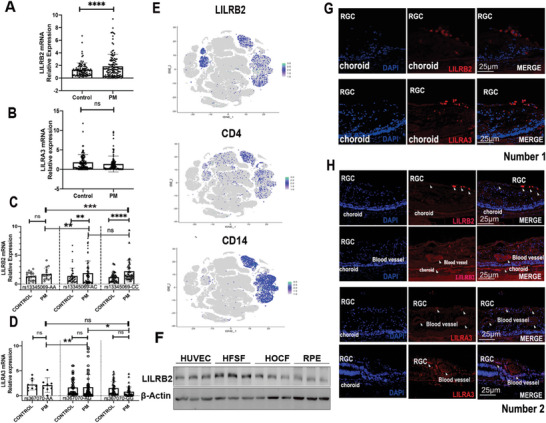
Expression of *LILRB2*/*LILRA3* in PM patients/controls and human eyes. A) This study enrolled 97 patients with pathological myopia (PM) and 126 control subjects and identified the association between the genotypes of SNPs and LILRB2/LILRA3. The expression of *LILRB2* was significantly higher in PM patients than in control subjects’ PBMC. B) The expression of *LILRA3* showed no significant differences between the groups (97 PM and 126 controls). C) After genotyping, the expression of *LILRB2* was significantly higher in PM cases carrying the rs13345069AC or rs13345069CC genotype (97 PM and 126 controls). In PM, the rs13345059C carriers had an increased risk of PM compared rs13345059A. D) The expression of *LILRA3* remained poorly associated with different genotypes of rs367070 (97 PM and 126 controls). In PM, the rs367070G carriers had an increased risk of PM compared rs367070A. E) In the single‐cell RNA sequencing of human tissue databases, we analyzed the expression of *LILRB2* and *LILRA3* genes. *LILRB2* is mainly expressed in CD4^+^ and CD14^+^ monocytes in human PBMCs. F) LILRB2 can be found in multiple important human ocular tissue–derived cell lines, namely human embryonic scleral fibroblasts (HFSFs), human umbilical vein endothelial cells (HUVECs), human ocular choroid fibroblasts (HOCFs), and retinal pigment epithelial (RPE) cells (*n* = 3). G,H) Both LILRB2 and LILRA3 proteins were mainly expressed in retinal blood vessels, choroid, and RGC cells in paraffin‐embedded sections of two human eye tissues. The expression of the two target proteins was not significant in human photoreceptor cells. All the *P* values were two‐sided. kD relative molecular weight in kilodalton. A two‐tailed Student's *t‐*test was conducted to assess statistical significance, ^*^ *p *< 0.05, ^**^ *p *< 0.01, ^***^ *p *<  0.001, ^****^ *p *<  0.0001.

To investigate the expression of *LILRB2* and *LILRA3* in human peripheral blood PBMCs, we enrolled 97 PM and 126 control subjects and collected their fresh peripheral blood for DNA and RNA extraction. The expression of *LILRB2* in PM cases was significantly higher than that in controls (**Figure** **2**A), whereas the expression of *LILRA3* in PM cases did not show a significant difference relative to controls (Figure 2B). As mentioned above, both rs13345059C and rs367070G carriers had an increased risk of PM compared with rs12517396A/rs367070A carriers (Table 2, OR < 1). After genotyping the DNA samples by standard PCR, we also found that *LILRB2* mRNA expression was significantly increased in PM cases carrying rs13345069AC/CC genotypes.

The rs13345054CC, which was completely linked to rs13345069CC, still showed higher expression of LILRB2 protein in the eQTL database in humans carrying this genotype (Figure S3A, Supporting Information). The individuals carrying the rs13345069CC genotype in the PM group exhibited the most pronounced genetic variances, as shown in Figure 2C. This finding implies that individuals with the rs13345069CC genotype may be more susceptible to increased expression of LILRB2. However, the *LILRA3* mRNA expression remained poorly associated with different genotypes of rs367070 (Figure 2D). A 6.7‐kb deletion in the *LILRA3* gene is widespread, and the frequency varies greatly between races. Such a huge deletion results in a sharp drop in protein expression levels. Therefore, the samples with all such deletions were excluded from this study, and expression differential analysis was performed again (Figure S4, Supporting Information). This analysis revealed that the expression of *LILRA3* was significantly different between PM and control cases. Specifically, in the population carrying rs367070GG, *LILRA3* expression was significantly downregulated in PM patients. The eQTL database also showed that individuals carrying the rs367070GG genotype had a lower expression of *LILRA*3 than those with the rs367070AA/AG genotype (Figure S3B, Supporting Information). The Hi‐C data also supports the regulation of *LILRB2* gene expression by the variant rs367070 (Figure S3C, Supporting Information).

### 2.6 Localization of LILRB2 and LILRA3 in Human Eyes

To investigate the expression of *LILRB2* and *LILRA3*, we first analyzed the expression of *LILRB2* and *LILRA3* in whole blood using single‐cell RNA sequencing data of normal human tissues.^[^ ^37^ ^]^ While *LILRA3* could not be detected in PBMC, *LILRB2* was mainly expressed in CD14^+^ monocytes (Figure 2E). Therefore, we focused on studying the *LILRB2* gene. We further performed protein expression analysis on four eye‐related cell lines, namely human embryonic scleral fibroblast (HFSF) cells, human ocular choroid fibroblasts (HOCF) and RPE. The human umbilical vein endothelial cells (HUVECs), which are frequently utilized in the context of ophthalmological research, are also included in this study. LILRB2 can obviously be found in all cell lines (Figure 2F). To further investigate the expression of LILRB2 and LILRA3 proteins in the human eye, we stained the paraffin‐embedded sections of two human eye tissues. Notably, both LILRB2 and LILRA3 proteins appeared in retinal vessels, the choroid, and the RGC (Figure 2G,H) in both human eyes.

### 2.7 Localization of LILRB2 and LILRA3 in Mouse Eyes

**Figure 3:**
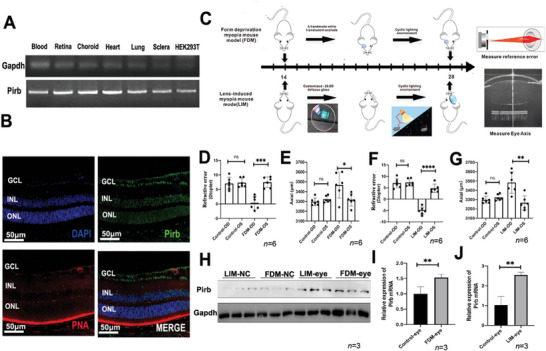
Expression of Pirb (LILRB2) in the eyes of mice and myopia mouse models. A) The expression of the orthologous gene of *LILRB2* in mice (Pirb) in the ocular tissue was investigated in mice by using reverse‐transcriptase polymerase chain reaction (RT‐PCR). The *LILRB2* (*Pirb*) gene was expressed in the blood, retina, choroid, heart, lung, and sclera of the eye in the mice. B) LILRB2 was widely expressed in the eye tissue of 2.5‐month‐old mice, especially in GCL and photoreceptor cells. C) Flowchart of the form‐deprivation myopia (FDM) and lens‐induced myopia (LIM) mouse models. D) The reference error results of the LDM mouse model (*n* = 6). E) Axial length of the LDM mouse mode (*n* = 6). F) The reference error results of the LIM mouse model (*n* = 6). G) Axial length of the LIM mouse model (*n* = 6). H) The retina of the control group and the experimental group was extracted, and the WB experiment showed that the expression of Pirb protein in the retina increased significantly (*n* = 3). I,J) The retina of the control group and the experimental group was extracted, and the qPCR experiment showed that the mRNA expression of Pirb in the retina increased significantly (*n* = 3). All the *P* values were two‐sided and adjustments were made for multiple comparisons. *n* indicates the number of biologically independent samples examined. The two‐tailed Student's *t*‐test and one‐way ANOVA were conducted to assess statistical significance, ^*^ *p *< 0.05, ^**^ *p *< 0.01, ^***^ *p *< 0.001.

We next investigated the expression of *LILRB2* orthologous genes (*Pirb*) in mice by reverse‐transcription polymerase chain reaction (RT‐PCR). The *Pirb* was expressed in the sclera, retina, and choroid of the eyes, as well as in the heart, lung, and blood (**Figure** **3**A). The frozen‐section analysis of 2.5‐month‐old mice's eyeballs showed similar results, that is, LILRB2/Pirb was widely expressed in eye tissues, especially in RGC, photoreceptor cells, and choroid (Figure 3B). PNA is a special marker for labeling cone cells. However, unlike human eyes, LILRB2 and LILRA3 proteins were not expressed in mouse photoreceptor cells.

### 2.8 Expression of Pirb in Myopia Mouse Models

Considering that the LILRA3 protein was not expressed in mice eyes, we mainly investigated the relationship between LILRB2 and myopia in a mouse model. In this study, the form‐deprivation myopia mouse model (FDM) and the lens‐induced myopia mouse model (LIM) were constructed using worn opaque eye patches and lenses to induce myopia in a single eye, respectively. The process and timeline for collecting experiment‐related parameters are shown in Figure 3C. This study determined the myopic representation of mice using refraction and axial measurement. The refractive error and AL measurement results of the two different myopia mice models are shown in Figure 3D–G.

The RT‐PCR and western blot (WB) results of the retina showed a significant difference in the expression of *Pirb* in the FDM and LIM groups compared with the matched control groups. Interestingly, both the mRNA and protein expression of *Pirb* showed a more significant increase in the LIM model eye (Figure 3H–J) than in the control eye (NC‐eye), suggesting that Pirb plays an important role in the formation of lens‐induced myopia in mice, and the expression of this protein increased significantly with the occurrence of myopia.

### 2.9 Overexpression of Pirb in Mouse Eyes Induces Myopia Phenotype

To evaluate the relationship between the high expression of *Pirb* and PM, we injected AAV8‐Pirb into the subretinal space in mice. The process and schedule of monocular injection of AAV into the mice are shown in Figure S5A. The results showed that only monocular injection of 1 µL AAV8‐*pirb* could cause mild myopia phenotype (Figure S5B–K, Supporting Information). Given that the effectiveness of binocular virus injection was more recognized, we subsequently conducted experiments using binocular injection.

**Figure 4:**
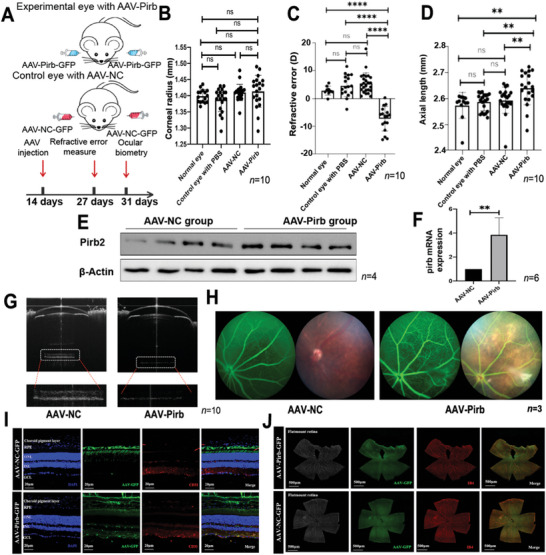
Overexpression of Pirb in mice causes PM. A) Flowchart of injecting different viruses into the mice. B) Corneal radius of the mice injected with different viruses. The results showed that the injection method used in this study did not significantly affect the curvature radius of the cornea and that it had a relatively small theoretical impact on subsequent overall refractive measurements (*n* = 10). C) The reference error results of the mice injected with different viruses. The results showed that injecting AAV‐Pirb into both eyes resulted in a significant decrease in the refractive index of the mice, indicating a shift in myopia refractive index (*n* = 10). D) Axial length results of the mice injected with different viruses. The results indicate that the injection of AAV‐Pirb into both eyes led to an increase in the length of the mice's eye axis (*n* = 10). E) The expression of Pirb protein was detected after bilateral injection of AAV‐Pirb and AAV‐NC (*n* = 4). F) The mRNA expression of Pirb was detected after bilateral injection of AAV‐Pirb and AAV‐NC (*n* = 6). G) Observing the ocular biological parameters of the mice using the OCT small‐animal ophthalmic biometric recognition system (VisonX, AOCT‐1000 M). After AAV‐Pirb injection, the ocular axis increased, and the retina (especially RPE‐choroid) became significantly thinner (*n* = 10). H) Fundus photography results of the mice injected with different viruses (*n* = 3). I,J) After AAV8 injection, it is mainly located near RPE and choroid (*n* = 3). All the p values were two‐sided and adjustments were made for multiple comparisons. *n* indicates the number of biologically independent samples examined. Scale bar, 20 and 500 µm. The two‐tailed Student's *t‐*test and one‐way ANOVA were conducted to assess statistical significance, ^*^ *p *< 0.05, ^**^ *p *< 0.01, ^***^ *p *< 0.001.

After injecting 1 µL AAV8‐*pirb* into the subretinal space of both eyes, the mice showed a pronounced myopia phenotype. The process and schedule of binocular injection are shown in **Figure** **4**A. To ensure the accuracy of the experiment, the corneal radius was measured in each mouse after injection to ensure that the intraocular‐injection in the study and the control group had no significant effect on the corneal radius of the mice (Figure 4B). The mice that were binocularly injected with AAV8‐*pirb* showed a significant decrease in refractive error (Figure 4C) and an increase in eye axis (Figure 4D) compared with those that were injected with AAV8‐NC in both eyes’ subretinal space. We further extracted the proteins and RNA from the mice's eyes and validated the overexpression of Pirb after the injection of AAV8‐*pirb* virus (Figure 4F). With the increase in the eye axis, the retina fundus of the mice became significantly thinner after the injection of AAV8‐*pirb* virus, making the mice's fundus present a phenotype similar to the human myopia‐related‐tessellation changes, which was characterized as a thinner RPE choroid pigment layer (Figure 4G,H). After AAV8 injection, it is mainly located near RPE and choroid (Figure 4I,J).

**Figure 5:**
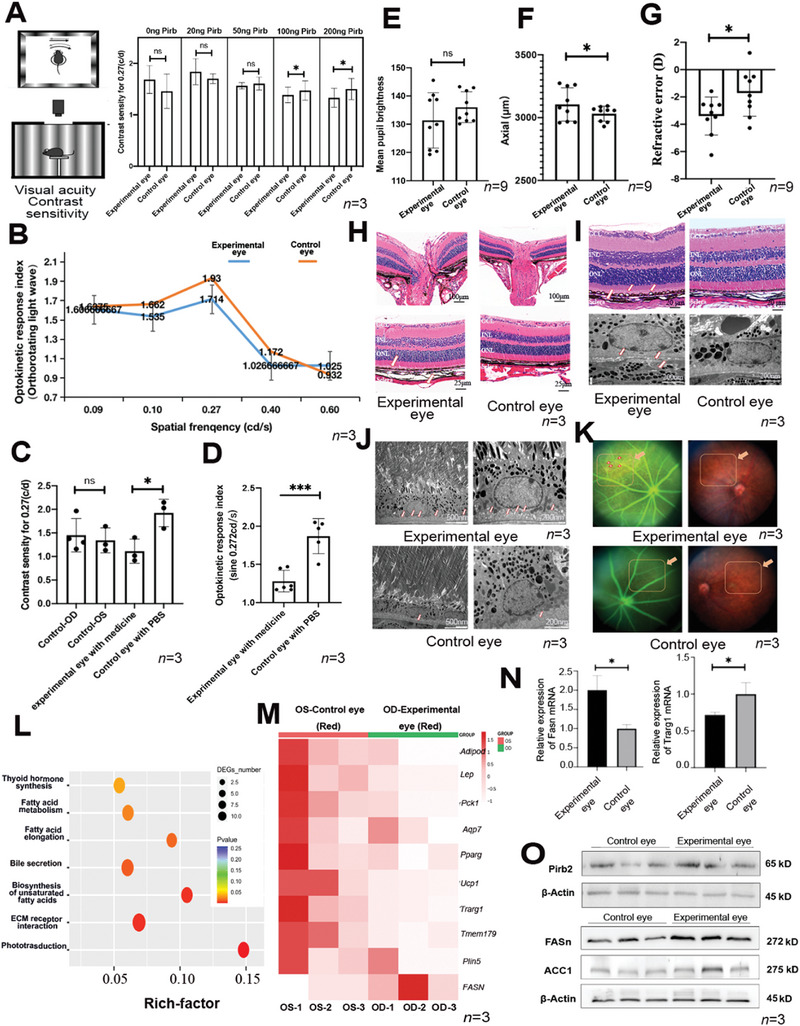
Increasing Pirb protein in mouse eyes induces significant changes in myopia phenotype. A) Injecting different concentrations of drugs into mice caused changes in their visual sensitivity (*n* = 3). B) Injecting a drug concentration of 200 ng into mice caused significant differences in their optokinetic response index (Orthorotating light wave) (*n* = 3). C) Injecting a drug concentration of 200 ng into mice caused a decrease in contrast density (*n* = 3). D) Injecting a drug concentration of 200 ng into mice caused a decrease in the optokinetic response index (*n* = 3). E) The pupil lightness results of the mice injected with 200 ng Pirb protein (*n* = 9). F) The axial length increased in the mice injected with 200 ng Pirb protein (*n* = 9). G) The reference error decreased in the mice injected with 200 ng Pirb protein (*n* = 9). H) Paraffin sectioning was performed on experimental mice, and HE staining was performed. Pathological changes were found near the retinal ganglia of the mice injected with 200 ng of the drug (*n* = 3). Scale bars, 100 and 25 µm. I) Significant suprachoroidal vacuoles were found near the choroid of the mouse fundus in the experimental group (*n* = 3). Scale bars, 20 µm, and 200 nm. J) After electron microscopic observation, it was found that small vacuoles also existed in the choroid, the area where lipids were abundant in the experimental group (*n* = 3). Scale bars, 500 and 200 µm. K) Fundus photography results of the mice injected with 200 ng of the drug. After taking fundus photos, we found that the experimental group exhibited a certain myopic arc‐shaped fundus phenotype (*n* = 3). L) Using RNA sequencing to analyze the difference between the experimental group and the control group, we found that the fatty acid metabolism pathway was significantly enriched. M) Differential analysis of the genes related to the fatty acid metabolism pathway in RNA sequencing (*n* = 3). N,O) The qPCR and WB results of fatty‐related gene expression (*n* = 3). The *P* values were two‐sided and adjustments were made for multiple comparisons. *n* indicates the number of biologically independent samples examined. The unpaired Student's *t*‐test and one‐way ANOVA were conducted to assess statistical significance, ^*^ *p *< 0.05, ^**^ *p *< 0.01, ^***^ *p *< 0.001.

To further verify the observation that overexpression of Pirb protein in mouse eyes (experimental eye) could induce myopia phenotype, we injected the soluble protein of the extracellular segment of Pirb into the subretinal space of the eye. We first injected soluble Pirb protein (20, 50, 100, and 200 ng; catalog number: 50760‐M08H) into the vitreous body of male mice for 2 weeks and then used the Opto‐Track animal visual inspection equipment to find the suitable drug concentration (**Figure** **5**A,B). This experiment indicated that the concentrations of 100 and 200 ng made a significant change in the refractive error (Figure 5C,D). We chose 200 ng Pirb as the final protein injection concentration. The experimental results showed that the increase in Pirb in the eye tissue did not affect the pupil diameter of the mice (Figure 5E) but led to a significant change in the refractive difference (Figure 5F) and the growth of the mouse eye axis (Figure 5G), thereby promoting myopia. Pathological changes were also found in the outer nuclear layer of the retina and the outer nuclear layer (Figure 5H) and choroidal cavities (Figure 5I,J).

### 2.10 Excessive Pirb Protein–Induced Lipid Accumulation in Mice Eyes

To explore the reasons for the observed phenomenon, we conducted RNA sequencing to determine the relevant differential signaling pathways in the whole posterior eye segment (Figure 5K). The analysis revealed that the pathways such as PPAR, cAMP, AMP, and fatty acid metabolism were significantly enriched. Due to the significant differences in the expression of proteins related to the fatty acid pathway, we further verified some genes by qPCR. The analysis confirmed that *Ucp‐1* and *Pck1* (fatty acid metabolism);^[^ ^41^ ^]^ *Fabp4*, *Adipoq*, and *Aqp7* (uptake, transport vectors)^[^ ^42^, ^43^ ^]^; *Scd‐1* (transformed proteins);^[^ ^44^ ^]^ and *Lep*, *Pparg*, and *Plin5* (fat decomposition)^[^ ^45^ ^]^ were significantly inhibited, indicating that the fatty acid metabolism pathways were inhibited (Figure 5L). Additionally, through quantitative polymerase chain reaction (qPCR) and western blot analysis, we confirmed that the Pirb peptide was capable of significantly upregulating Pirb protein expression in murine ocular tissues and inducing an elevation in lipid‐related proteins (Figure 5M–O). The RNA‐seq results are shown in Supporting Information.

**Figure 6:**
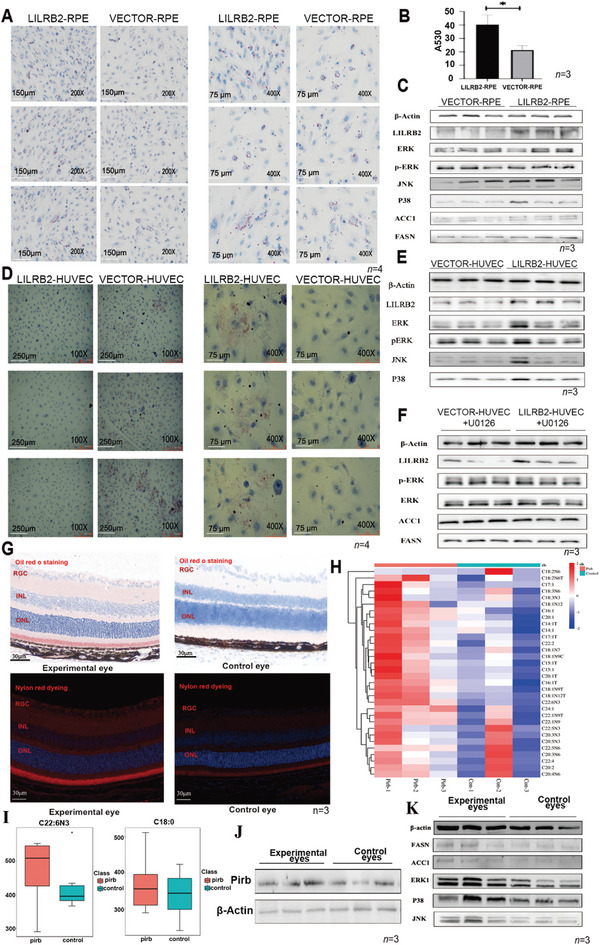
An increase in Pirb protein causes the accumulation of lipids in cells and tissues. A) Oil‐red‐O staining was used to detect the difference in fat droplet formation in RPE cells. Overexpression of LILRB2 led to an increase in fat droplets in RPE cells. Scale bars, 150 and 75 µm. B) Statistical analysis of oil‐red‐O staining test results. C) Overexpression of LILRB2 increased the expression of FASN and ACC1, and the ERK1–P38–JNK pathway was also activated in RPE cells. D) Oil‐red‐O staining was used to detect the difference in fat droplet formation in HUVEC. Overexpression of LILRB2 led to an increase in fat droplets in HUVEC. Scale bars, 250 and 75 µm. E) Overexpression of LILRB2 activated the ERK1–P38–JNK pathway in HUVEC cells. F) After inhibiting the ERK signaling pathway, the expression of ACC1 and FASN proteins was no longer increased. U0126, the known ERK1/2 inhibitor. G) We used oil‐red‐O‐staining and Nile‐red‐fluorescence staining on mouse eye tissues and found the accumulation of triglycerides in the retina/choroid of the Pirb‐injected mice. Scale bar, 30 µm. H,I) Accumulation of medium‐ and long‐chain fatty acids in the retina/choroid of the mice injected with Pirb. J,K) WB results of the ERK1–P38–JNK pathway and two fatty acid synthases in the experimental eye. The *P* values were two‐sided and adjustments were made for multiple comparisons. An unpaired two‐tailed Student's *t*‐test was conducted to assess statistical significance, ^*^ *p *< 0.05, ^**^ *p *< 0.01, and ^***^ *p *< 0.001.

RPE and HUVEC cell lines are valuable tools for investigating choroidal oxygen supply.^[^ ^43^, ^44^ ^]^ Given the previous findings that LILRB2 was expressed in RPE and HUVEC, we constructed the LILRB2‐Flag plasmid and transferred it into RPE and HUVEC. After transfection of the plasmids, there was a significant increase in fat droplets after overexpression of LILRB2, as revealed using oil‐red‐O staining. The next experiments suggested that the ERK–P38–JNK pathway was activated by LILRB2 overexpression in both RPE and HUVEC (**Figure** **6**A–E), whereas an ERK1/2 inhibitor (U0126) did not exert similar effects (Figure 6F).

We further conducted oil‐red‐O‐staining and Nile‐red‐fluorescence staining on mouse eye tissues and found the accumulation of triglycerides in the retina/choroid of the Pirb‐injected mice (Figure 6G). The accumulation of medium‐ and long‐chain fatty acids in the retina/choroid of the Pirb‐injected mice was also found by further metabolomic analysis, indicating that the accumulation of fatty acids might lead to histopathological changes in Pirb‐rich condition (Figure 6H,I). We also found a significant increase in the ERK1–P38–JNK pathway and fatty acid synthase (FASN and ACC1), which indirectly demonstrated a significant change in the fatty acid pathway (Figure 6J,K).

Furthermore, previous studies have indicated a correlation between LILRB2 proteins and lipid deposition within signaling pathways including ERK1–JNK–P38 and cAMP45. Consequently, we conducted in vitro experiments to confirm this signaling pathway. The collective findings support the notion that overexpression of LILRB2 leads to lipid deposition through the activation of the ERK1‐JNK‐P38 signaling pathway, resulting in increased expression of *FASN* and *ACC1* genes associated with fat synthesis. Other results details are shown in the Supporting information.

## 3 Discussion

Both genetic and environmental factors contribute to the development of myopia; however, the genetic influence is considered more important for PM than for other types of myopia. It is expected that the Asian adult population will still be at a high risk of developing PM in the next 100 years.^[^ ^48^ ^]^ GWAS is a highly recognized research method that has identified several associated loci for a myopia‐associated phenotype. Our GWAS identified one novel locus (19q13.42) and one previously identified refractive error locus (15q25.1)^[^ ^39^, ^49^ ^]^ as newly associated loci for PM. Although a recent genetic study has had a larger sample size, it only investigated complex traits with higher prevalence, such as refractive error.^[^ ^50^ ^]^ In contrast, recruitment of patients with PM is difficult due to its lower prevalence, particularly those with degenerative changes (namely degenerative myopia). To improve detection power, we assigned PM patients with longer axis (greater than 26.0 mm) to the first stage. This strategy might be the reason why we were successful in identifying the candidate region with a relatively small number of cases.

15q25 (*RASGRF1*) has been associated with refractive error,^[^ ^39^ ^]^ and high myopia in East Asians.^[^ ^51^, ^52^ ^]^ We further identified its association with PM (rs745030‐*RASGRF1*, 15q25.1) in East Asians. *RASGRF1* encodes Ras protein‐specific guanine nucleotide releasing factor 1. The protein encoded by this gene is a guanine nucleotide exchange factor (GEF). Functional analysis has demonstrated that this protein stimulates the dissociation of GDP from RAS protein. Rasgrf1 upregulation has been found in the sclera of myopic eyes; however, further investigation is needed to determine whether Rasgrf1 plays a causative role or whether it is a consequence of myopia‐induced scleral remodeling.^[^ ^53^ ^]^

This study revealed that locus rs367070‐*LILRA3*/rs13345069‐*LILRB2* (19q13.42) is associated with PM (*P* = 2.63 × 10^−22^ and OR = 0.68 for rs367070; *P* = 2.47 × 10^−12^ and OR = 0.68 for rs13345069). *LILRA3* and *LILRB2* encode two members of the immunoglobulin‐like receptor (LIR) family.^[^ ^54^ ^]^ Previous studies have indicated that the monomeric *LILRA3* is independently expressed in the nucleus^[^ ^55^ ^]^ and *LILRB2* is widely expressed in cell lines. LILRB2 contains two or four extracellular immunoglobulin domains, a transmembrane domain, and 2–4 cone cytoplasmic immunoreceptor tyrosine‐based inhibitory motifs (ITIMs).^[^ ^56^, ^57^, ^58^ ^]^ The murine homolog of LILRB2 appears to be paired immunoglobulin‐like receptor‐B (Pirb), the only inhibitory form of the murine‐paired immunoglobulin‐like receptor family concerning expression pattern, ligands, and function. There is a high degree of similarity in all of the amino acid sequences, protein structures, and expression patterns of LILRB2 and Pirb protein (Figure S6, Supporting Information).

Limited research has been conducted on the expression and function of LILRA3 and LILRB2 in ocular tissues. In this study, we detected *LILRA3* expression in blood and *LILRB2* expression might be located in human eye tissues (retina, RGC, RPE, blood vessels, and choroid) and cell lines (HFSF, HUVEC, HOCF, and RPE). The genotypes of rs13345059 were significantly associated with the expression of *LILRB2*, and *LILRB2* expression was significantly increased in carriers of rs13345059C, who had a higher risk of developing PM. Further, cellular and animal experimental evidence is required to establish whether the presence of the rs13345059C carriers can directly elicit alterations in the LILRB2 protein. *LILRA3* was significantly less expressed in PM compared with controls in PBMC, especially in the population carrying rs367070GG. The eQTL and sQTL data of the GTEx Portal database showed that SNP rs367070‐*LILRA3* more significantly affected the expression of LILRB2 in the whole blood. *LILRB2* alleles are in linkage disequilibrium with *LILRA3* deletion polymorphism, and the region shows evidence of positive selection.^[^ ^59^ ^]^ Therefore, rs367070‐*LILRA3* may participate in the process of PM by regulating the expression of LILRB2, suggesting that LILRB2 may be more closely related to PM.

Previous studies have shown that LILRB2 can bind with the neuron proliferation inhibitory protein (Nogo, RTN4), a family of endoplasmic reticulum membrane proteins that have been identified as axon‐growth inhibitory proteins of the central nervous system,^[^ ^60^ ^]^ and ameliorate lipid accumulation in tissues.^[^ ^33^, ^61^ ^]^ The existence of the Nogo protein can affect the dynamic visual and OKR impairment of mice.^[^ ^35^ ^]^ LILRB2 is a high‐affinity receptor for Nogo‐A protein,^[^ ^62^ ^]^ and the reduction of Nogo protein can effectively regulate the occurrence of obesity, suggesting that they may also affect the function of fat synthesis.^[^ ^33^, ^61^ ^]^

The experimental results of both FDM and LIM mouse myopia models showed that the expression of Pirb protein increased in myopia eyes, and it increased more significantly in the LIM myopia mice. Combined with the increased expression of LILRB2 in PM patients, we used fundus injection of AAV‐Pirb and injection of extracellular functional peptide segments to detect the relationship between this gene and PM. The experimental eyes that overexpressed Pirb using AAV exhibited significant myopia refractive drift. Subsequently, given that the extracellular segment of Pirb is the core region of its function, our injected Pirb experiment increased the ocular axis, decreased the refractive power, led to choroidal cavern pathological changes, and caused lipid accumulation.

The choroidal cavern is a new fundus feature proposed in 2016, which can significantly affect normal functions, such as the supply of oxygen to the choroid membrane.^[^ ^63^, ^64^, ^65^ ^]^ Hypoxia of the sclera tissue induced by choroidal oxygen supply can lead to a decrease in the elasticity of collagen tissue in the sclera, weaken the adjustment of the ocular axis, and promote the formation of pathological myopia.

The metabolism of an eye, especially photoreceptors, also requires lipid substances. Genes, lipoproteins, and lipases required for lipid metabolism are not expressed in the outer retina but are highly expressed in the choroid membrane.^[^ ^64^ ^]^ This study revealed that the accumulation of lipids induced by abnormal lipid metabolism in the eye tissue may also be an important driving factor for myopia. Some studies have pointed out that adiponectin, which plays an important role in lipid metabolism, can promote fatty acid oxidation and inhibit lipid synthesis.^[^ ^66^ ^]^ In addition, the level of adiponectin always negatively correlates with the occurrence of myopia.^[^ ^67^ ^]^

Transcriptome analysis of the posterior sclera and choroid (not retina) of Pirb‐injected mice showed a significant decrease in PPAR, CAMP, and other proteins related to fat metabolism, indicating significant disturbances in fat synthesis and metabolism‐related pathways. By comparing the metabolomic determination of free long‐chain fatty acids in Pirb‐injected eyes with those in normal eyes, we found that the utilization rate of functional free fatty acids in the injected eyes was lower, which also confirmed the disorder of fatty acid metabolism. Finally, both RPE and HUVEC are important tool cell lines for studying choroidal oxygen supply.^[^ ^46^, ^47^ ^]^ The conducted cell experiments demonstrate that upregulation of the full‐length fragment of LILRB2 led to the activation of the ERK1/JNK/P38 signaling pathway, whereas an ERK1/2 inhibitor (U0126) did not have a similar effect, resulting in elevated expression of FASN and ACC1 proteins and subsequent augmentation of lipid droplet formation in RPE and HUVEC. These findings further validate the association between elevated LILRB2 expression in ocular tissues and the development of aberrant ocular manifestations, including lipid accumulation and choroidal indentation, which compromise choroidal function, impair ocular self‐regulation, and heighten the susceptibility to PM.

**Figure 7:**
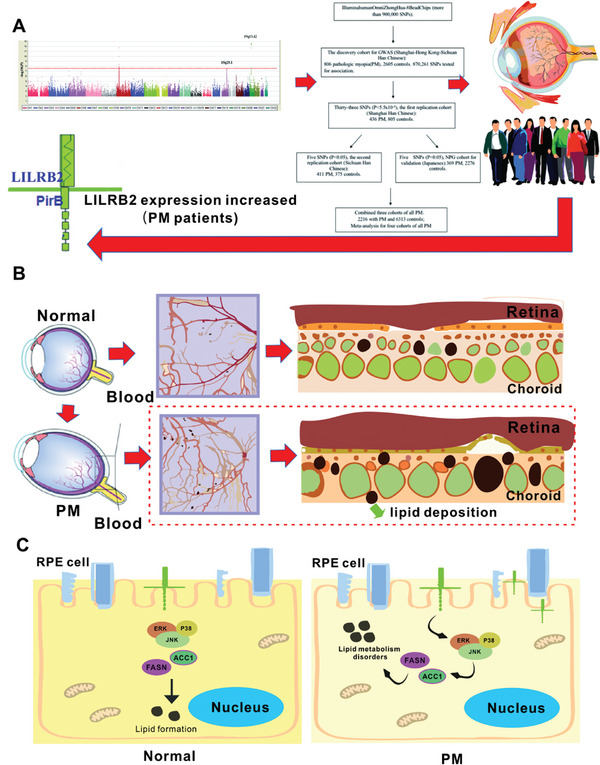
Potential pathogenic mechanisms of LILRB2. A) We identified *LILRB2* could be an important candidate gene for PM. B) LILRB2 increases fatty acid synthesis and lipid accumulation in RPE/choroid in different mouse models. C) In cell lines, the lipid accumulation due to overexpression of LILRB2 could be mediated via the activation of ERK1/JNK/P38 signaling, resulting in choroid caverns and worse visual acuity, destroying the function of the choroid and promoting the development of PM.

The GWAS conducted in this research has inherent limitations. Primarily, it was only able to offer a relative risk prediction range rather than precise individual and genetic pathogenesis predictions. Consequently, the first half of this study represented an initial investigation into the pathogenesis of PM, and further exploration of associated mechanisms is needed. Unlike the previous GWAS, our study has identified LILRB2/LILRA3 as novel genes associated with PM and investigated their role in the pathogenesis of this condition. Specifically, we found that upregulation of LILRB2, due to genetic factors, leads to enhanced fatty acid synthesis and lipid accumulation in RPE/choroid through the activation of the ERK1/JNK/P38 signaling pathway. This process results in the formation of choroidal caverns, deterioration of visual acuity, impairment of choroidal function, and progression of PM, as depicted in **Figure** **7**.

## 4 Experimental Section

### Study Subjects

Ethical approval for human research was granted by the Institutional Ethics Committee of the Medical College of Sichuan Provincial People's Hospital under protocol number 2021–465. Animal experiments were conducted with approval from the same committee under protocol number 2018–057. All procedures used in this study conformed to the tenets of the Declaration of Helsinki. The participants were recruited and fully informed of the purpose and procedures, and written informed consent was obtained from each participant. The subjects underwent comprehensive ophthalmologic investigations by ophthalmologists, including dilated indirect ophthalmoscopy and contact lens slit‐lamp biomicroscopy, automatic objective refraction evaluation, and measurement of the AL by applanation A‐scan ultrasonography (UD‐6000, Tomey, Nagoya, Japan) or partial coherence interferometry (IOLMaster, Carl Zeiss Meditec, Dublin, CA, USA). Eligible participants met the criteria for bilateral onset of myopia with an AL exceeding 26 mm and a spherical equivalent less than 8.0 D. The exclusion criteria included the presence of glaucoma, cataracts, spherical lens, keratoconus, other corneal diseases, or systemic connective tissue diseases. Healthy volunteers were recruited concurrently to serve as normal controls, and they met the criteria of diopter levels within ±0.75 D for at least 1 year and an eye AL of 24 mm.^[^ ^2^ ^]^ This is a two‐stage study, including discovery stage and replication stage. The discovery stage cohort is from Sichuan, including 806 patients and 2591 controls. In the replication stage, three cohorts are included: the replication cohort 1 was from Shanghai (436 patients, 805 controls), cohort 2 was from Guangzhou (411 patients, 375 controls), and cohort 3 was from Japan (466 patients, 2262 controls). The clinical data of the study participants are presented in Table 1. In addition, to investigate the expression of *LILRB2* and *LILRA3* in human peripheral blood mononuclear cells (PBMCs), we enrolled 97 individuals with PM and 126 control subjects and collected their fresh peripheral blood for DNA and RNA extraction. None of the 97 patients and 126 controls had any immunological comorbidity and they were all immunocompetent.

### Genome‐Wide Association Analysis

For the discovery‐stage analysis, a total of 900015 single‐nucleotide polymorphisms (SNPs) were genotyped in 806 patients with PM and 2591 control cases using Illumina Human Omni Zhonghua‐8 BeadChip (Illumina, San Diego, CA, USA). This chip specifically covers the rare variants found in the Chinese population. It covers ≈81% of the common variants in the Chinese population (r^2^ > 0.8), and the minor genotype frequency (MAF) is more than 5%. For rare variants with MAF > 2.5%, the chip covers ≈60%. The chip adopts the beam array technology patented by Illumina, which can improve the detection quality of the chip. The average detection rate is more than 99%, and the repetition rate is more than 99.9%. Cluster definition for each SNP was performed using Illumina BeadStudio Genotyping Module. A systematic quality‐control procedure of the genome scan results was applied as follows. We filtered samples using a call rate of 95%, meaning that we retained SNPs for which there was less than 5% missing data. We removed SNPs with a MAF less than 1%. Samples with an inbreeding coefficient greater than 0.1, calculated as |*F*| = (1 − *O*/*E*), were removed, where *O* and *E* represent the observed and expected counts of heterozygous SNPs within an individual, respectively.^[^ ^21^ ^]^ We removed SNPs with a Hardy–Weinberg equilibrium (HWE) test *p* value of less than 1 × 10^−6^ in controls. Samples were evaluated for data quality first, and markers were subsequently excluded (806 PM cases and 2591 controls were used for the following data analysis).

After whole‐genome imputation, principal‐component analysis was performed to remove samples with outlying ancestry from further analysis using the R statistical software package (SNPRelate, snpgdsPCA).^[^ ^22^, ^23^ ^]^ Then, we examined potential genetic relatedness based on pairwise identity by state for all of the successfully genotyped samples using PLINK 1.9 software.^[^ ^24^ ^]^ The genomic inflation estimate (λGC) was calculated for variants with MAF > 0.5% as λGC = 1.005 using only directly genotyped SNPs by PLINK. Single‐marker association analyses were performed by PLINK 1.9, adjusted for sex, the average age at which patients were diagnosed with PM, and top 10 principal components for population stratification.

### Genotype Imputation

Genotypes were converted to the PLINK binary format, and we excluded SNPs with missingness > 10%, MAF < 1%, and HWE p < 10^−6^ for phasing. Then, clean data were phased using SHAPEIT2.^[^ ^25^ ^]^ After that, the dataset was imputed with 1000 Genomes Phase 1 (version 3) of CHB and CHS (hg19) on MiniMac3 website.^[^ ^26^ ^]^

After imputation, a quality‐control step was performed to filter imputed data with high degrees of uncertainty. An *R* ^2^ threshold of 0.7 was applied for the inclusion in association analysis, where *R* ^2^ in this context represents the correlation coefficient determined by a linear model regressing each imputed SNP on regional typed SNPs.^[^ ^21^, ^27^ ^]^ At this stage, SNPs with MAF less than 0.01 were excluded. In total, 7817399 SNPs were used for association analysis. Single‐marker association analyses were performed by PLINK 1.9, adjusted for sex, reporting mean age, and top 10 principal components for population stratification.^[^ ^24^ ^]^ A Bonferroni‐corrected genome‐wide significance threshold of 5 × 10^−8^ was used for control of the family‐wise error rate.

### SNP Selection for Replication Studies

Three SNPs showing association with PM surpassing *P* ≤ 9 × 10^−8^ in the GWAS discovery stage were brought forward to the replication stage and analyzed using the same method as in the discovery stage. Two reported SNPs in the 15q25.1 were also selected. Five SNPs were selected for replication analysis. All primers required for these experiments are shown in Table S1 (Supporting Information).

### Genotyping and Quality Controls in the Replication Studies

Genotyping analysis of the SNPs selected for the first replication was conducted using the Sequenom Mass ARRAY system as previously described. The association analysis of the replication genotype data was conducted using PLINK 1.9,^[^ ^24^ ^]^ adjusted for sex and age. In the first replication, a total of 436 cases and 805 controls were tested; in the second replication, 411 cases and 375 controls were tested; and in the third replication, 466 cases and 2262 controls were tested.

### Meta‐Analysis and MAGMA Gene‐Set Analysis

We used the PLINK 1.9 software to perform a combined meta‐analysis of the GWAS discovery and replication datasets for PM. MAGMA was used as a tool for gene analysis and generalized gene‐set analysis of the GWAS data of the discovery stage. The summary SNP p values from the GWAS data were used for analysis in accordance with the protocol.

### RT‐PCR to Detect the Content of LILRA3 and LILRB2 in Humans and Mice

Total RNA was reverse‐transcribed to complementary DNA (cDNA) (1 µg in a final volume of 20 µL) using Invitrogen SuperScript cDNA synthesis Kit as recommended by the manufacturer (Thermo Fisher) for RNA extraction from tissues. The primers are listed in Table S1 (Supporting Information).

### Induction of Monocular form‐Deprivation Myopia Mouse Model (FDM) and Lens‐Induced Myopia Mouse Model (LIM) in Mice

FDM, a mouse myopia model, was induced by pasting a handmade white translucent hemispherical piece of plastic containing polyethylene material on the right eye.^[^ ^28^ ^]^ LIM, another mouse myopia model, was induced by a defocused glass of −10.0 D (customized by Beijing Jing De Jia Run Vision Co., Ltd.). The procedure of FDM/LIM induction has been previously described in detail.^[^ ^28^ ^]^ To prevent the occluder from being removed by the mice, a thin plastic collar was fitted around the mice's neck for 7 days. Normal control (NC) mice were not fitted with diffuser goggles or a plastic collar around the neck.

### Adeno‐associated Virus (AAV) Vector Construction and Virus Injection

AAV8‐Pirb was acquired from Shanghai Jikai Co., Ltd., China. Approximately 0.5 µL/1 µL AAV8 and AAV8‐Pirb virus supernatant (10^13^ genome copies mL^−1^) were injected into the subretinal space of the mice, using a pulled angled glass pipette under direct observation aided by a dissecting microscope under dim light.^[^ ^29^ ^]^

### Ocular Biometric Measurements

Infrared auto‐refractometry (Photorefractor; STRIA.tech; developed by Frank Schaeffel, Germany) was used to measure the refraction error of the mice. The small‐animal infrared keratometer (STRIA.tech; developed by Professor Frank Schaeffel) was used to measure the corneal radius and curvature in the mice.^[^ ^30^ ^]^ By analyzing the reflection points of eight infrared LEDs on the corneal surface, the corneal curvature radius of conscious small animals can be automatically and conveniently measured with this keratometer equipment. In different groups, the mice's eyes were anesthetized using the OCT Biometric System for Small Animal Ophthalmology (VisonX, AOCT‐1000 M) to facilitate measurement of the eye axis.^[^ ^31^ ^]^ The OCT device of this anterior segment is mainly used for eye axis detection. The fundus of the mice was observed by a small‐animal fundus imaging system. For better observation, sodium fluorescein was administered via intraperitoneal injection in this study to label the retinal, choroid, and other eye vessels.

### Measurements of Spatial Frequency and Contrast Sensitivity

Young (P14–P21) male C57BL/6 Wild‐type (WT) mice were used for behavioral vision measurements and protein injection. The body weight of the mice ranged from 9 to 11 g. Then, the Pirb extracellular segment protein (soluble PirB ectodomain) was injected into the right eye of each mouse by intravitreal injection. Soluble PirB outer domain protein (sPirB) has been considered a potential therapeutic approach for diseases. Injecting this type of protein can significantly increase its expression in a short period of time.^[^ ^29^, ^32^, ^33^ ^]^ After 2 weeks, the spatial frequency and contrast sensitivity thresholds of the optokinetic response (OKR) were measured with the virtual optomotor system in freely moving mice with Opto‐Trak (Dynamic Tracking System for Visual Stimulation of Small Animals, XR‐OT101).^[^ ^34^, ^35^ ^]^ The OKR spatial‐frequency sensitivity threshold was determined by presenting full‐contrast gratings with increasing spatial frequency (starting from 0.27 cycle/degree [c/d]), until the maximum frequency that the mice were able to discriminate was reached. Contrast thresholds were determined at six spatial frequencies (0.009, 0.10, 0.27, 0.40, 0.192, and 0.60 c/d) by decreasing the contrast of the gratings until the tracking behavior ceased. Soluble Pirb protein (20, 50, 100, and 200 ng; catalog number: 50760‐M08H) was injected into the vitreous body of male mice for 2 weeks and then used the Opto‐Track animal visual inspection equipment to find the suitable drug concentration. Finally, it was found that the concentrations of 100 and 200 ng yielded the best results. Therefore, the protein concentration of 200 ng was used as the final experimental concentration.

### Cell Culture and Transfection Assay

HUVEC and ARPE19 cells were grown as monolayer cultures in ECM and DMEM/F12 supplemented with 10% fetal bovine serum and antibiotics (purchased from ATCC). To establish transfected cell lines overexpressing *LILRB2*, a plasmid encoding LILRB2 in pcDNA3.1 (purchased from Youbao biology) was transfected into human RPE and HUVEC cells using a lip3000 transfection reagent (Invitrogen).

### Western Blot

Cells and tissues were extracted in 150 µL cell lysis buffer (CST, USA) containing proteinase inhibitors (Bio Tools, USA). The lysates were centrifuged at 12 000 rpm at 4 °C for 15 min, and the supernatants were collected for western blot analysis. The protein concentration of the supernatants was determined by the BCA protein assay kit. The samples were separated on 10% SDS‐polyacrylamide gel and transferred to nitrocellulose membranes. The membranes were incubated with a monoclonal antibody at 4 °C overnight. Next, they were incubated with horseradish peroxidase (HRP)–labeled secondary antibodies (1:2000, CST, USA) at room temperature for 1 h. The membranes were detected by automatic exposure.

### Statistical Analysis

The statistical analysis methods in GWAS have been detailed in the preceding sections. The Bonferonni‐corrected significance threshold of 0.05 was used to control the error rate. Clinical data were expressed as the mean ± standard error of the mean (SEM). All experiments were repeated at least three times to ensure reproducible outcomes. Statistical analysis was conducted with GraphPad Prism 8.0 package. Preliminary assessments were conducted on the data sets to assess normality utilizing the Shapiro–Wilk test. Nonparametric tests were utilized in instances where the data did not adhere to a normal distribution. Statistical differences between distinct experimental groups were typically assessed using a Student's *t‐*test or one‐way/two‐way ANOVA for normally distributed data. A P‐value threshold of less than 0.05 was deemed indicative of statistical significance. The mean fluorescence intensities and protein grayscale were computed using the integrated “Analyze‐Measure” tool in ImageJ. Subsequent statistical analysis was then carried out using Prism 8 (GraphPad).

## Conflict of Interest

The authors declare no conflict of interest.

## Ethics Approval

The human samples and animal studies in this study were approved and supervised by the Institutional Ethics Committee of the Medical College of Sichuan Provincial People's Hospital (No. 2021‐465 and 2018–057). This study conformed with the Helsinki Declaration of 1975 (as revised in 2008) concerning Human and Animal Rights, and they followed the policy concerning Informed Consent as shown on Springer.com.

## Author Contributions

L.J. and L.H. contributed equally to this work. Y.S., Z.Y., and L.H. were involved in the study conception and design. L.J., R.Z., and C.D. performed the experiments. H.L., Q. L., and W.R. made interpreted the data. L.J. wrote the original manuscript. Z.Y. and Y.‐B. M. revised the manuscript. M.M., Y.M., S.‐ya N., K.M., and K.Y. provided DNA samples. All authors read and approved the final manuscript.

## Supporting information

Supporting Information

Supporting Information

Supporting Information

## Data Availability

The data that support the findings of this study are available from the corresponding author upon reasonable request.

## References

[1] K. Ohno‐Matsui , J. B. Jonas , Prog. Retin Eye Res. 2019, 70, 99. 30537538 10.1016/j.preteyeres.2018.12.001

[2] J. B. Jonas , R. A. Jonas , M. M. Bikbov , Y. X. Wang , S. Panda‐Jonas , Prog. Retin Eye Res. 2023, 96, 101156. 36585290 10.1016/j.preteyeres.2022.101156

[3] Y. Fang , T. Ishida , R. Du , S. Xie , T. Igarashi‐Yokoi , T. Yoshida , T. Watanabe , Y. Onishi , K. Ohno‐Matsui , Ophthalmology 2021, 128, 477. 32730954 10.1016/j.ophtha.2020.07.044

[4] E. Moisseiev , G. Yiu , Lancet 2017, 389, 1133. 27817867 10.1016/S0140-6736(16)31407-6

[5] Y. Wang , S. Chen , J. Lin , W. Chen , H. Huang , X. Fan , X. Cao , M. Shen , J. Ye , S. Zhu , A. Xue , F. Lu , Y. Shao , Invest. Ophthalmol. Vis. Sci. 2022, 63, 20. 10.1167/iovs.63.12.20 PMC9672896 36378132

[6] J. Ye , M. Wang , M. Shen , S. Huang , A. Xue , J. Lin , Y. Fan , J. Wang , F. Lu , Y. Shao , Invest. Ophthalmol. Vis. Sci. 2020, 61, 45. 10.1167/iovs.61.4.45 PMC7401930 32343783

[7] Z. Zhang , Y. Xu , J. Liu , D. W. Wong , C. K. Kwoh , S. M. Saw , T. Y. Wong , PLoS One 2013, 8, e65736. 23799040 10.1371/journal.pone.0065736 PMC3683061

[8] M. Schwartz , M. Haim , D. Skarsholm , Clin. Genet. 1990, 38, 281. 1980096

[9] T. L. Young , S. M. Ronan , A. B. Alvear , S. C. Wildenberg , W. S. Oetting , L. D. Atwood , D. J. Wilkin , R. A. King , Am. J. Hum. Genet. 1998, 63, 1419. 9792869 10.1086/302111 PMC1377552

[10] T. L. Young , S. M. Ronan , L. A. Drahozal , S. C. Wildenberg , A. B. Alvear , W. S. Oetting , L. D. Atwood , D. J. Wilkin , R. A. King , Am. J. Hum. Genet. 1998, 63, 109. 9634508 10.1086/301907 PMC1377231

[11] P. Paluru , S. M. Ronan , E. Heon , M. Devoto , S. C. Wildenberg , G. Scavello , A. Holleschau , O. Makitie , W. G. Cole , R. A. King , T. L. Young , Invest. Ophthalmol. Vis. Sci. 2003, 44, 1830. 12714612 10.1167/iovs.02-0697

[12] P. C. Paluru , S. Nallasamy , M. Devoto , E. F. Rappaport , T. L. Young , Invest. Ophthalmol. Vis. Sci. 2005, 46, 2300. 15980214 10.1167/iovs.04-1423

[13] Q. Zhang , X. Guo , X. Xiao , X. Jia , S. Li , J. F. Hejtmancik , Mol. Vis. 2005, 11, 554. 16052171

[14] Q. Zhang , X. Guo , X. Xiao , X. Jia , S. Li , J. F. Hejtmancik , J. Med. Genet. 2006, 43, e20. 16648373 10.1136/jmg.2005.037853 PMC2564525

[15] S. Nallasamy , P. C. Paluru , M. Devoto , N. F. Wasserman , J. Zhou , T. L. Yong , Mol. Vis. 2007, 13, 229. 17327828 PMC2633468

[16] C. Y. Lam , P. O. Tam , D. S. Fan , B. J. Fan , D. Y. Wang , C. W. Lee , C. P. Pang , D. S. Lam , Invest. Ophthalmol. Vis. Sci. 2008, 49, 3768. 18421076 10.1167/iovs.07-1126

[17] S. Paget , S. Julia , Z. G. Vitezica , V. Soler , F. Malecaze , P. Calvas , Mol. Vis. 2008, 14, 2566. 19122830 PMC2613077

[18] H. Nakanishi , R. Yamada , N. Gotoh , H. Hayashi , K. Yamashiro , N. Shimada , K. Ohno‐Matsui , M. Mochizuki , M. Saito , T. Iida , K. Matsuo , K. Tajima , N. Yoshimura , F. Matsuda , PLoS Genet. 2009, 5, e1000660. 19779542 10.1371/journal.pgen.1000660 PMC2735651

[19] C. C. Khor , M. Miyake , L. J. Chen , Y. Shi , V. A. Barathi , F. Qiao , I. Nakata , K. Yamashiro , X. Zhou , P. O. Tam , C. Y. Cheng , E. S. Tai , E. N. Vithana , T. Aung , Y. Y. Teo , T. Y. Wong , M. Moriyama , K. Ohno‐Matsui , M. Mochizuki , F. Matsuda , R. Y. Yong , E. P. Yap , Z. Yang , C. P. Pang , S. M. Saw , N. Yoshimura , Hum. Mol. Genet. 2013, 22, 5288. 23933737 10.1093/hmg/ddt385

[20] F. Zhao , D. Zhang , Q. Zhou , F. Zhao , M. He , Z. Yang , Y. Su , Y. Zhai , J. Yan , G. Zhang , A. Xue , J. Tang , X. Han , Y. Shi , Y. Zhu , T. Liu , W. Zhuang , L. Huang , Y. Hong , D. Wu , Y. Li , Q. Lu , W. Chen , S. Jiao , Q. Wang , N. Srinivasalu , Y. Wen , C. Zeng , J. Qu , X. Zhou , EBioMedicine 2020, 57, 102878. 32652319 10.1016/j.ebiom.2020.102878 PMC7348000

[21] E. Reed , S. Nunez , D. Kulp , J. Qian , M. P. Reilly , A. S. Foulkes , Stat. Med. 2015, 34, 3769. 26343929 10.1002/sim.6605 PMC5019244

[22] K. J. Galinsky , G. Bhatia , P. R. Loh , S. Georgiev , S. Mukherjee , N. J. Patterson , A. L. Price , Am. J. Hum. Genet. 2016, 98, 456. 26924531 10.1016/j.ajhg.2015.12.022 PMC4827102

[23] N. Patterson , A. L. Price , D. Reich , PLoS Genet. 2006, 2, e190. 17194218 10.1371/journal.pgen.0020190 PMC1713260

[24] S. Purcell , B. Neale , K. Todd‐Brown , L. Thomas , M. A. Ferreira , D. Bender , J. Maller , P. Sklar , I. de Bakker P , M. J. Daly , P. C. Sham , Am. J. Hum. Genet. 2007, 81, 559. 17701901 10.1086/519795 PMC1950838

[25] O. Delaneau , J. F. Zagury , J. Marchini , Nat. Methods 2013, 10, 5. 23269371 10.1038/nmeth.2307

[26] S. Das , L. Forer , S. Schonherr , C. Sidore , A. E. Locke , A. Kwong , S. I. Vrieze , E. Y. Chew , S. Levy , M. McGue , D. Schlessinger , D. Stambolian , P. R. Loh , W. G. Iacono , A. Swaroop , L. J. Scott , F. Cucca , F. Kronenberg , M. Boehnke , G. R. Abecasis , C. Fuchsberger , Nat. Genet. 2016, 48, 1284. 27571263 10.1038/ng.3656 PMC5157836

[27] T. W. Winkler , F. R. Day , D. C. Croteau‐Chonka , A. R. Wood , A. E. Locke , R. Magi , T. Ferreira , T. Fall , M. Graff , A. E. Justice , J. Luan , S. Gustafsson , J. C. Randall , S. Vedantam , T. Workalemahu , T. O. Kilpelainen , A. Scherag , T. Esko , Z. Kutalik , I. M. Heid , R. J. Loos , Nat. Protoc. 2014, 9, 1192. 24762786 10.1038/nprot.2014.071 PMC4083217

[28] K. W. Qian , Y. Y. Li , X. H. Wu , X. Gong , A. L. Liu , W. H. Chen , Z. Yang , L. J. Cui , Y. F. Liu , Y. Y. Ma , C. X. Yu , F. Huang , Q. Wang , X. Zhou , J. Qu , Y. M. Zhong , X. L. Yang , S. J. Weng , Neurosci. Bull 2022, 38, 992. 35349094 10.1007/s12264-022-00842-9 PMC9468212

[29] D. N. Bochner , R. W. Sapp , J. D. Adelson , S. Zhang , H. Lee , M. Djurisic , J. Syken , Y. Dan , C. Shatz , J. Sci. Transl. Med. 2014, 6, 140. 10.1126/scitranslmed.3010157 PMC4476552 25320232

[30] F. Schaeffel , Front. Biosci. 2008, 13, 4904. 18508555 10.2741/3049

[31] H. Ni , S. Xu , L. Tian , J. Mao , J. Li , N. Lin , P. Hu , Z. Wu , X. Chen , Z. Bao , J. Zheng , P. Yan , R. Deng , BMC Med. Imaging 2023, 23, 194. 37990166 10.1186/s12880-023-01147-7 PMC10664477

[32] B. Deng , L. Li , X. Gou , H. Xu , Z. Zhao , Q. Wang , L. Xu , Mol. Neurobiol. 2018, 55, 652. 27987133 10.1007/s12035-016-0301-9

[33] D. P. Li , L. Huang , R. R. Kan , X. Y. Meng , S. Y. Wang , H. J. Zou , Y. M. Guo , P. Q. Luo , L. M. Pan , Y. X. Xiang , B. B. Mao , Y. Y. Xie , Z. H. Wang , M. Yang , R. He , Y. Yang , Z. L. Liu , J. H. Xie , D. L. Ma , B. P. Zhang , S. Y. Shao , X. Chen , S. M. Xu , W. T. He , W. J. Li , Y. Chen , X. F. Yu , Nat. Commun. 2023, 14, 4436. 37481670 10.1038/s41467-023-40183-3 PMC10363120

[34] H. Qin , W. Zhang , S. Zhang , Y. Feng , W. Xu , J. Qi , Q. Zhang , C. Xu , S. Liu , J. Zhang , Y. Lei , W. Liu , S. Feng , J. Wang , X. Fu , Z. Xu , P. Li , K. Yao , J. Exp. Med. 2023, 220. 10.1084/jem.20220776 PMC10037108 36930174

[35] A. Guzik‐Kornacka , A. van der Bourg , F. Vajda , S. Joly , F. Christ , M. E. Schwab , V. Pernet , Brain Struct. Funct. 2016, 221, 317. 25284126 10.1007/s00429-014-0909-3

[36] S. M. Tang , F. F. Li , S. Y. Lu , K. W. Kam , P. Tam , C. C. Tham , C. P. Pang , J. Yam , L. J. Chen , Br. J. Ophthalmol. 2020, 104, 1472. 31300455 10.1136/bjophthalmol-2019-314203

[37] H. Zhu , J. Chen , K. Liu , L. Gao , H. Wu , L. Ma , J. Zhou , Z. Liu , J. J. Han , Sci. Adv. 2023, 9, q7599. 10.1126/sciadv.abq7599 PMC10306289 37379396

[38] M. Oishi , K. Yamashiro , M. Miyake , Y. Akagi‐Kurashige , K. Kumagai , I. Nakata , H. Nakanishi , M. Yoshikawa , A. Oishi , N. Gotoh , A. Tsujikawa , R. Yamada , F. Matsuda , N. Yoshimura , Invest. Ophthalmol. Vis. Sci. 2013, 54, 7492. 24150758 10.1167/iovs.13-12825

[39] P. G. Hysi , T. L. Young , D. A. Mackey , T. Andrew , A. Fernandez‐Medarde , A. M. Solouki , A. W. Hewitt , S. Macgregor , J. R. Vingerling , Y. J. Li , M. K. Ikram , L. Y. Fai , P. C. Sham , L. Manyes , A. Porteros , M. C. Lopes , F. Carbonaro , S. J. Fahy , N. G. Martin , M. van Duijn C , T. D. Spector , J. S. Rahi , E. Santos , C. C. Klaver , C. J. Hammond , Nat. Genet. 2010, 42, 902. 20835236 10.1038/ng.664 PMC4115148

[40] W. R. Liu , J. Kim , C. Nwankwo , L. K. Ashworth , Immunogenetics. 2000, 51, 659. 10941837 10.1007/s002510000183

[41] P. Cohen , S. Kajimura , Nat. Rev. Mol. Cell Biol. 2021, 22, 393. 33758402 10.1038/s41580-021-00350-0 PMC8159882

[42] J. Deng , Y. Guo , F. Yuan , S. Chen , H. Yin , X. Jiang , F. Jiao , F. Wang , H. Ji , G. Hu , H. Ying , Y. Chen , Q. Zhai , F. Xiao , F. Guo , Autophagy 2020, 16, 451. 31184563 10.1080/15548627.2019.1628537 PMC6999619

[43] M. Quattrocelli , M. Wintzinger , K. Miz , M. Panta , A. D. Prabakaran , G. D. Barish , N. S. Chandel , E. M. McNally , J. Exp. Med. 2022, 219. 10.1084/jem.20211906 PMC8980841 35363257

[44] V. Ratziu , L. de Guevara , R. Safadi , F. Poordad , F. Fuster , J. Flores‐Figueroa , M. Arrese , A. L. Fracanzani , B. D. Ben , K. Lackner , T. Gorfine , S. Kadosh , R. Oren , M. Halperin , L. Hayardeny , R. Loomba , S. Friedman , A. Sanyal , J. Nat. Med. 2021, 27, 1825. 10.1038/s41591-021-01495-3 PMC12165723 34621052

[45] V. I. Gallardo‐Montejano , C. Yang , L. Hahner , J. L. McAfee , J. A. Johnson , W. L. Holland , R. Fernandez‐Valdivia , P. E. Bickel , Nat. Commun. 2021, 12, 3320. 34083525 10.1038/s41467-021-23601-2 PMC8175597

[46] P. Sehgal , S. Mathew , A. Sivadas , A. Ray , J. Tanwar , S. Vishwakarma , G. Ranjan , K. V. Shamsudheen , R. C. Bhoyar , A. Pateria , E. Leonard , M. Lalwani , A. Vats , R. R. Pappuru , M. Tyagi , S. Jakati , S. Sengupta , K. B. B. , S. Chakrabarti , I. Kaur , R. K. Motiani , V. Scaria , S. Sivasubbu , EMBO J. 2021, 40, 107134. 10.15252/embj.2020107134 PMC8327952 34180064

[47] J. Song , K. Lee , S. W. Park , H. Chung , D. Jung , Y. R. Na , H. Quan , C. S. Cho , J. H. Che , J. H. Kim , J. H. Park , S. H. Seok , Invest. Ophthalmol. Vis. Sci. 2018, 59, 3747. 30046816 10.1167/iovs.18-23892

[48] I. G. Morgan , K. Ohno‐Matsui , S. M. Saw , Lancet 2012, 379, 1739. 22559900 10.1016/S0140-6736(12)60272-4

[49] V. J. Verhoeven , P. G. Hysi , R. Wojciechowski , Q. Fan , J. A. Guggenheim , R. Hohn , S. MacGregor , A. W. Hewitt , A. Nag , C. Y. Cheng , E. Yonova‐Doing , X. Zhou , M. K. Ikram , G. H. Buitendijk , G. McMahon , J. P. Kemp , B. S. Pourcain , C. L. Simpson , K. M. Makela , T. Lehtimaki , M. Kahonen , A. D. Paterson , S. M. Hosseini , H. S. Wong , L. Xu , J. B. Jonas , O. Parssinen , J. Wedenoja , S. P. Yip , D. W. Ho , et al., Nat. Genet. 2013, 45, 314. 23396134 10.1038/ng.2554 PMC3740568

[50] M. S. Tedja , R. Wojciechowski , P. G. Hysi , N. Eriksson , N. A. Furlotte , V. Verhoeven , A. I. Iglesias , M. A. Meester‐Smoor , S. W. Tompson , Q. Fan , A. P. Khawaja , C. Y. Cheng , R. Hohn , K. Yamashiro , A. Wenocur , C. Grazal , T. Haller , A. Metspalu , J. Wedenoja , J. B. Jonas , Y. X. Wang , J. Xie , P. Mitchell , P. J. Foster , B. Klein , R. Klein , A. D. Paterson , S. M. Hosseini , R. L. Shah , C. Williams , et al., Nat. Genet. 2018, 50, 834. 29808027 10.1038/s41588-018-0127-7 PMC5980758

[51] H. Hayashi , K. Yamashiro , H. Nakanishi , I. Nakata , Y. Kurashige , A. Tsujikawa , M. Moriyama , K. Ohno‐Matsui , M. Mochizuki , M. Ozaki , R. Yamada , F. Matsuda , N. Yoshimura , Invest. Ophthalmol. Vis. Sci. 2011, 52, 4853. 21436269 10.1167/iovs.11-7311

[52] X. Jiao , P. Wang , S. Li , A. Li , X. Guo , Q. Zhang , J. F. Hejtmancik , Mol. Vis. 2012, 18, 2633. 23170057 PMC3501279

[53] Q. Gong , M. Janowski , M. Xie , G. Yang , L. Liu , Clin. Exp. Optom. 2017, 100, 174. 27723119 10.1111/cxo.12476

[54] M. Wang , M. Liu , J. Jia , H. Shi , J. Teng , H. Liu , Y. Sun , X. Cheng , J. Ye , Y. Su , H. Chi , T. Liu , Z. Wang , L. Wan , J. Meng , Y. Ma , C. Yang , Q. Hu , Arthritis Rheumatol. 2021, 73, 1033. 33381895 10.1002/art.41635 PMC8252061

[55] H. An , A. Richardson , P. Rajasekariah , L. Zhong , B. Fernando , A. Macmillan , E. Klotzsch , K. Bryant , N. O. Kaakoush , N. Tedla , J. Proteome Res. 2021, 20, 3078. 33793249 10.1021/acs.jproteome.0c00946

[56] N. A. Fanger , D. Cosman , L. Peterson , S. C. Braddy , C. R. Maliszewski , L. Borges , Eur. J. Immunol. 1998, 28, 3423. 9842885 10.1002/(SICI)1521-4141(199811)28:11<3423::AID-IMMU3423>3.0.CO;2-2

[57] J. Zheng , M. Umikawa , C. Cui , J. Li , X. Chen , C. Zhang , H. Huynh , X. Kang , R. Silvany , X. Wan , J. Ye , A. P. Canto , S. H. Chen , H. Y. Wang , E. S. Ward , C. C. Zhang , Nature 2012, 485, 656. 22660330 10.1038/nature11095 PMC3367397

[58] M. Colonna , J. Samaridis , M. Cella , L. Angman , R. L. Allen , C. A. O'Callaghan , R. Dunbar , G. S. Ogg , V. Cerundolo , A. Rolink , J. Immunol. 1998, 160, 3096. 9531263

[59] K. Hirayasu , J. Ohashi , H. Tanaka , K. Kashiwase , A. Ogawa , M. Takanashi , M. Satake , G. J. Jia , N. O. Chimge , E. W. Sideltseva , K. Tokunaga , T. Yabe , Am. J. Hum. Genet. 2008, 82, 1075. 18439545 10.1016/j.ajhg.2008.03.012 PMC2427302

[60] K. C. Wang , V. Koprivica , J. A. Kim , R. Sivasankaran , Y. Guo , R. L. Neve , Z. He , Nature 2002, 417, 941. 12068310 10.1038/nature00867

[61] X. Wang , Y. Yang , D. Zhao , S. Zhang , Y. Chen , Y. Chen , K. Feng , X. Li , J. Han , Y. Iwakiri , Y. Duan , X. Yang , J. Biol. Chem. 2022, 298, 101561. 34998825 10.1016/j.jbc.2022.101561 PMC8814669

[62] H. An , M. Brettle , T. Lee , B. Heng , C. K. Lim , G. J. Guillemin , M. S. Lord , E. Klotzsch , C. L. Geczy , K. Bryant , T. Fath , N. Tedla , J. Cell Sci. 2016, 129, 1198. 26826187 10.1242/jcs.182006

[63] D. P. Mucciolo , D. Giorgio , M. Lippera , V. Dattilo , I. Passerini , E. Pelo , A. Sodi , G. Virgili , F. Giansanti , V. Murro , Invest. Ophthalmol. Vis. Sci. 2022, 63, 25. 10.1167/iovs.63.2.25 PMC8857610 35156991

[64] R. Dolz‐Marco , J. P. Glover , O. Gal‐Or , K. M. Litts , J. D. Messinger , Y. Zhang , M. Cozzi , M. Pellegrini , K. B. Freund , G. Staurenghi , C. A. Curcio , Ophthalmology. 2018, 125, 1287. 29625839 10.1016/j.ophtha.2018.02.036 PMC6321740

[65] S. R. Singh , M. Lupidi , S. B. Mishra , M. Paez‐Escamilla , G. Querques , J. Chhablani , Surv Ophthalmol 2020, 65, 451. 31978382 10.1016/j.survophthal.2020.01.001

[66] I. Vasiliauskaite‐Brooks , R. Sounier , P. Rochaix , G. Bellot , M. Fortier , F. Hoh , L. De Colibus , C. Bechara , E. M. Saied , C. Arenz , C. Leyrat , S. Granier , Nature 2017, 544, 120. 28329765 10.1038/nature21714 PMC5595237

[67] F. F. Li , M. C. Zhu , Y. L. Shao , F. Lu , Q. Y. Yi , X. F. Huang , Invest. Ophthalmol. Vis. Sci. 2023, 64, 7. 10.1167/iovs.64.3.7 PMC9988699 36867130

