# Supplementary material for: Genome‐Wide Association Analysis Identifies LILRB2 Gene for Pathological Myopia
Source: Adv Sci (Weinh). 2024 Aug 29;11(40):2308968. doi: 10.1002/advs.202308968 (PMC11516067; doi:10.1002/advs.202308968)
Supplement: Supplementary file 1 — Supporting Information [file ADVS-11-2308968-s001.docx]

**Supplementary Figures and Legends**


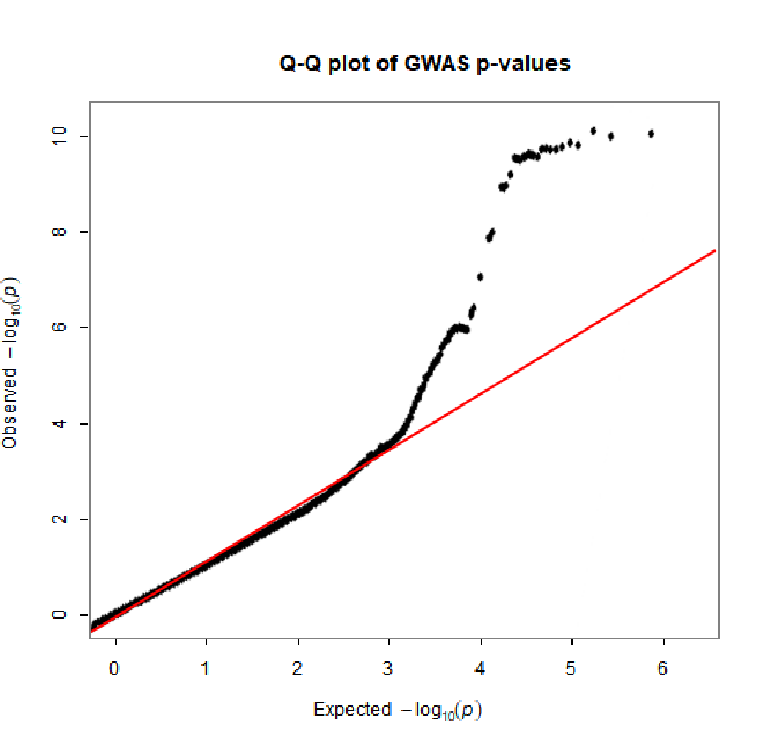


**Figure S1: Quantile–quantile plots of the observed *P* values (–log_10_*P*) for association.**

The genomic inflation factor (λ) in the genome-wide association analysis (GWAS) was 1.016.


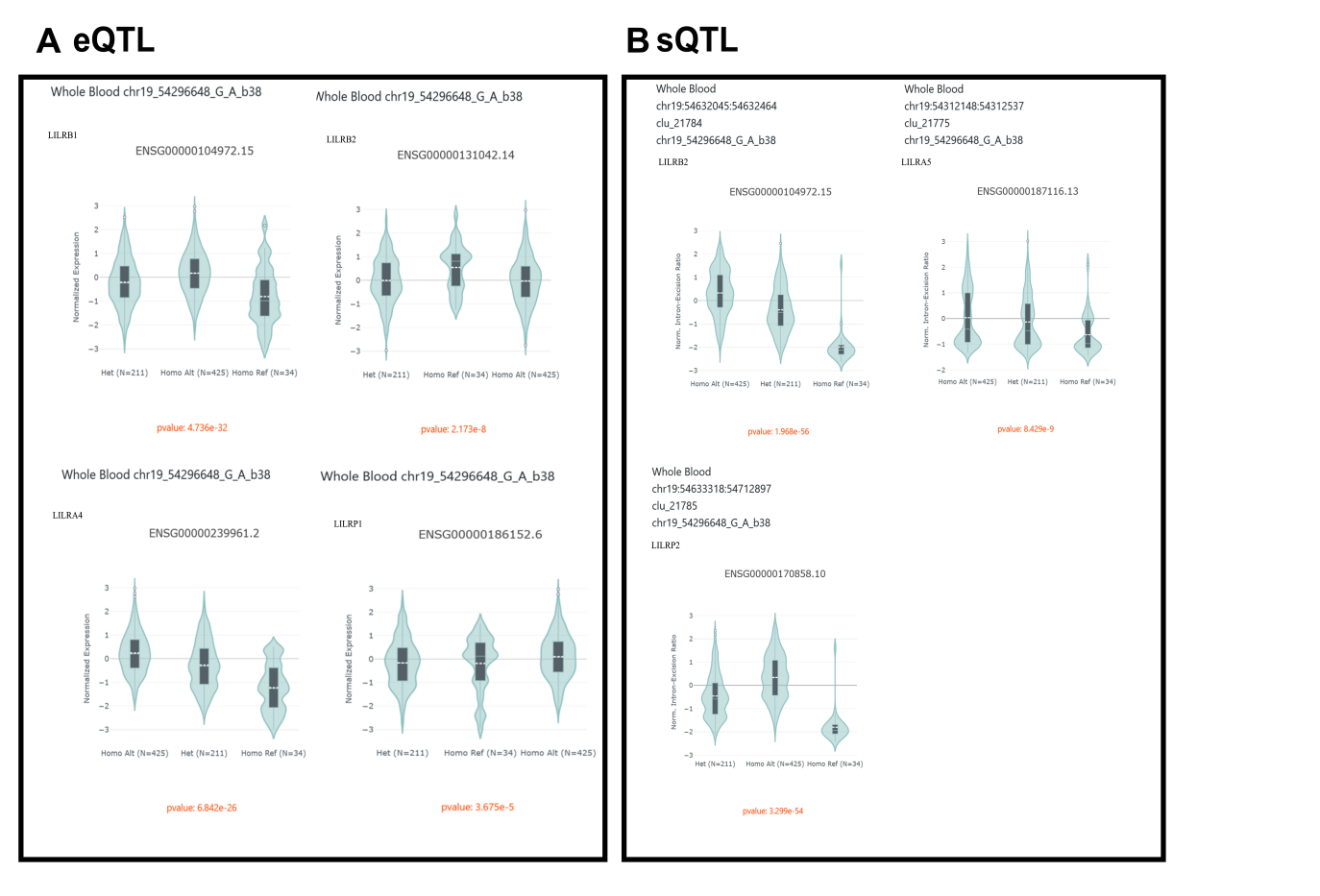


**Figure S2: The eQTL and sQTL data of rs367070-*LILRA3* locus in GTEX.**

**(A–B)**, The eQTL and sQTL data of GTEX showed that rs367070-*LILRA3* locus affects the expression of most leukocyte immunoglobulin-like receptor (ILR) family members (e.g., *LILRB1*, *LILRP2*, *LILRA4*), but it does not significantly affect the expression of *LILRA3*. Both results of eQTL and sQTL suggest that the rs367070-*LILRA3* locus may have a more significant effect on the function of the *LILRB2* gene than that of the *LILRA3* gene.


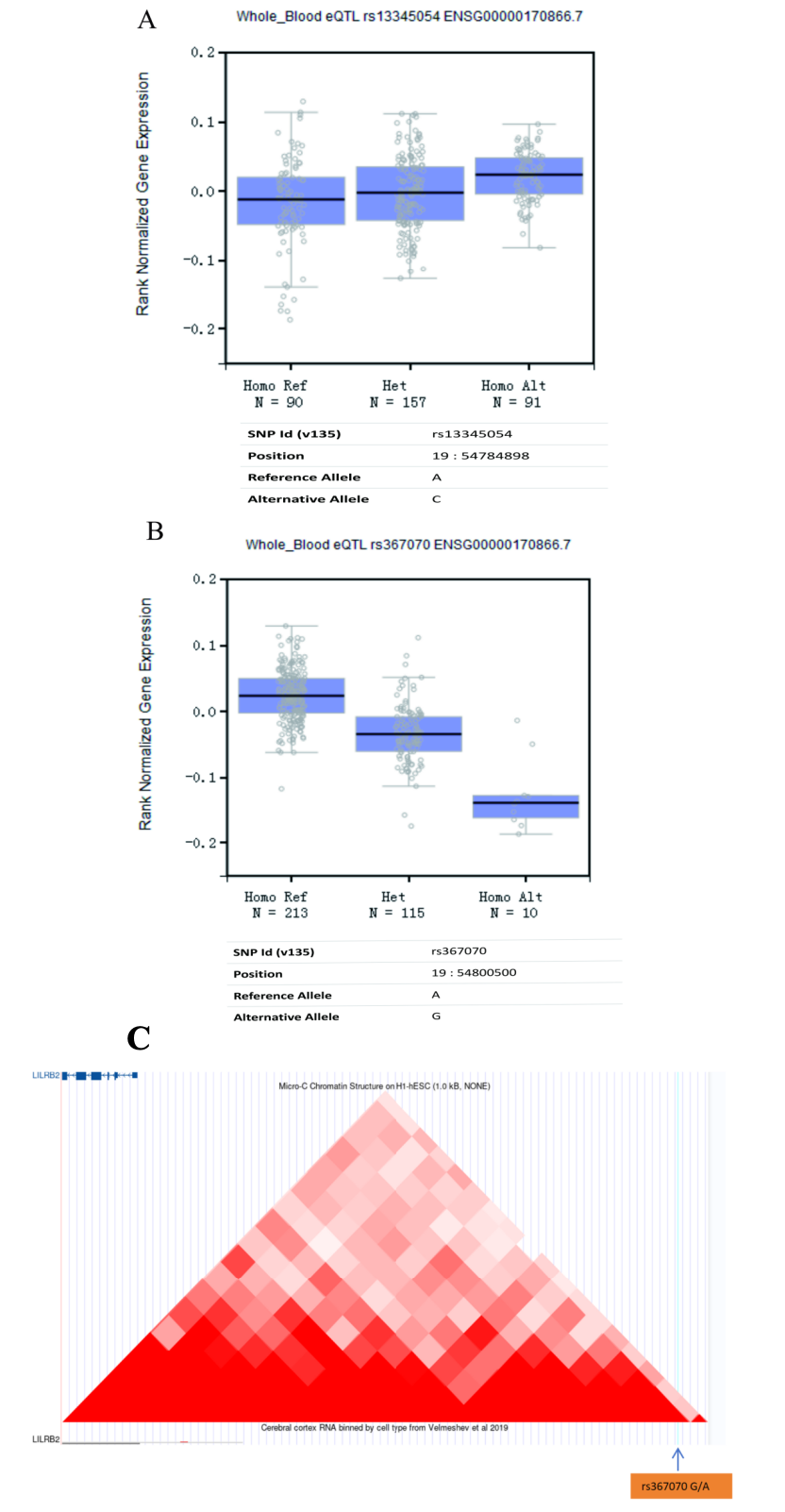


**Figure S3: Differences in gene expression of SNPs (rs13345054 and rs367070) in whole blood from eQTL (from https://www.gtexportal.org/) and Hi-C data (from https://genome.ucsc.edu/).**

The eQTL database also shows differences in the expression of *LILRB2* and *LILRA3* in individuals carrying different genotypes. Hi-C data also supports the regulation of *LILRB2* gene expression by the variant rs367070.


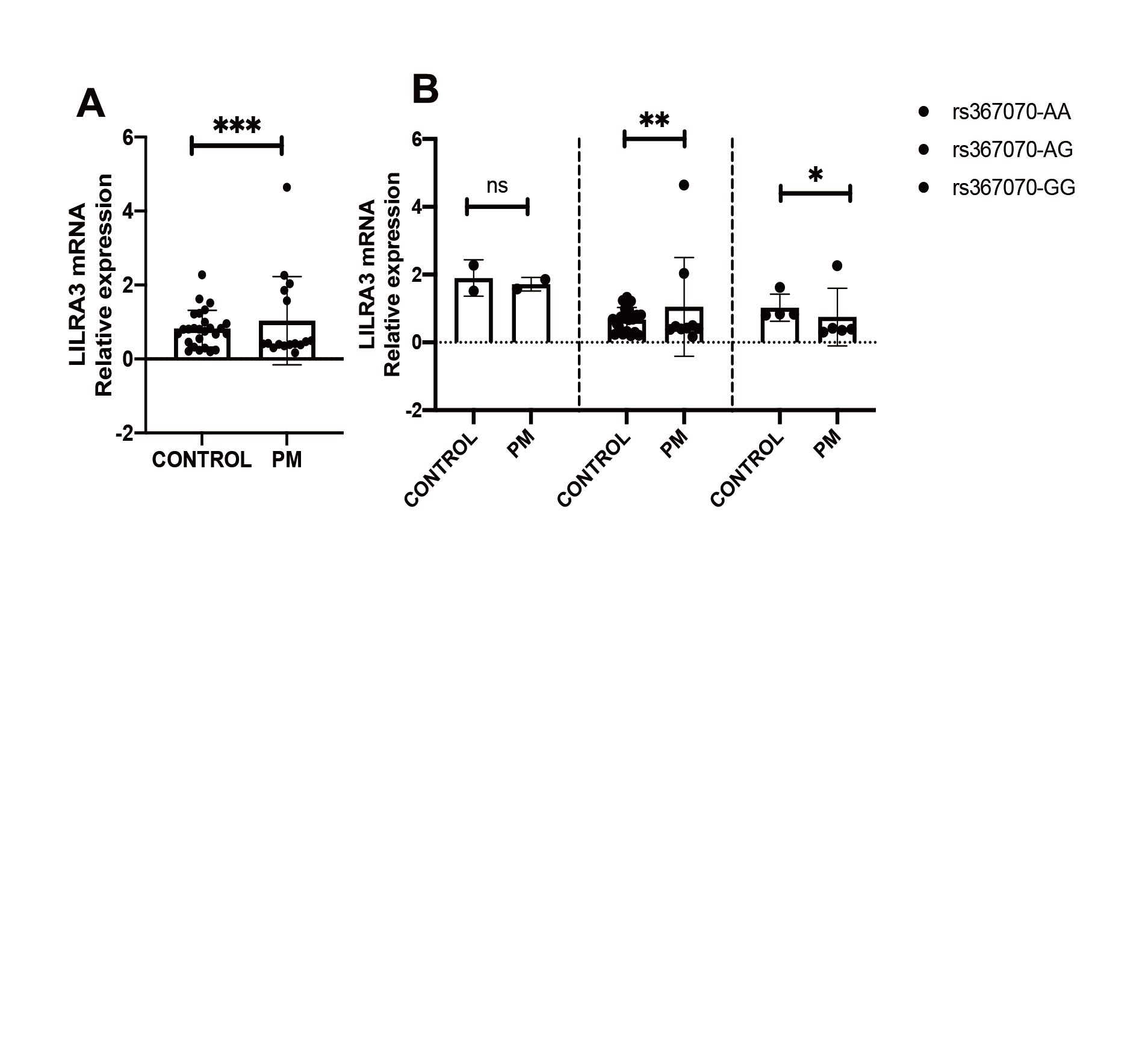


**Figure S4: Comparison of the association between different SNP-genotypes and LILRA3 gene expression in patients and normal individuals.**

**(A**) Gene expression differential analysis of *LILRA3* between PM and controls. In the evolutionary process, there is a 6.7-kb deletion in the *LILRA3* gene of many people. Such a long-segment gene deletion not only significantly affects gene expression but also leads to functional changes in the corresponding protein of the gene. Patients with *LILRA3* gene deletion were not excluded, and there was no significant difference in gene expression between PM and control. (**B**) The expression of *LILRA3* excluding people carrying 6.7-kb deletion in the *LILRA3* gene. PM patients carrying rs367070AG/GG showed higher expression of the *LILRA3* gene than PM patients carrying rs367070AA (*: *P* < 0.05, ** *P* < 0.01,***: *P* < 0.001).


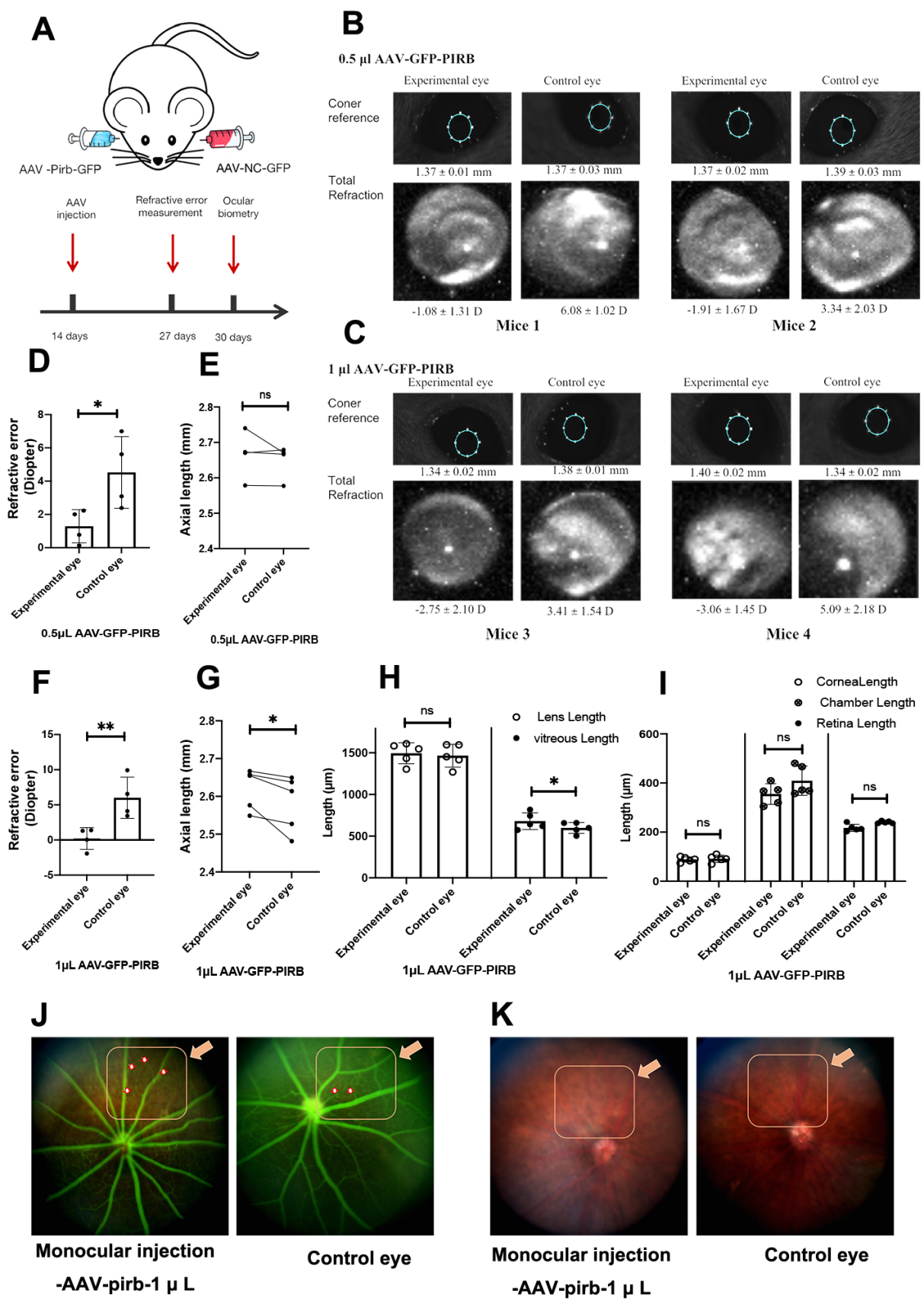


**Figure S5:** **Overexpression of Pirb (LILRB2) in mouse monocular causes myopia.**

**(A)** The process and schedule of monocular injection of AAV into mice subretinal space. (**B)** Changes in reference error after monocular injection of 0.5 μL AAV-Pirb. (**C**) Changes in reference error after monocular injection of 1 μL AAV-Pirb. (**D**) Changes in refractive error after monocular injection of 0.5 μL AAV-Pirb (n=4). (**E**) Changes in AL after monocular injection of 0.5 μL AAV-Pirb (n=4). (**F**) Changes in refractive error after monocular injection of 0.5 μL AAV-Pirb (n=5). (**G)** Changes in lens length after monocular injection of 1 μL AAV-Pirb (n=5). (**H)** Changes in lens length after monocular injection of 1 μL AAV-Pirb (n=5). (**I**) Thickness changes of the three refractive media (cornea, chamber, and retina) after monocular injection of 1 μL AAV-Pirb (n=5). (**J–K**) AAV Pirb injection can cause slight changes in the fundus of the eye. The changes in the fundus of the eye are mainly reflected in a slight increase in the end swelling of the blood vessels (indicated by arrows). The *P* values were two-sided and adjustments were made for multiple comparisons.


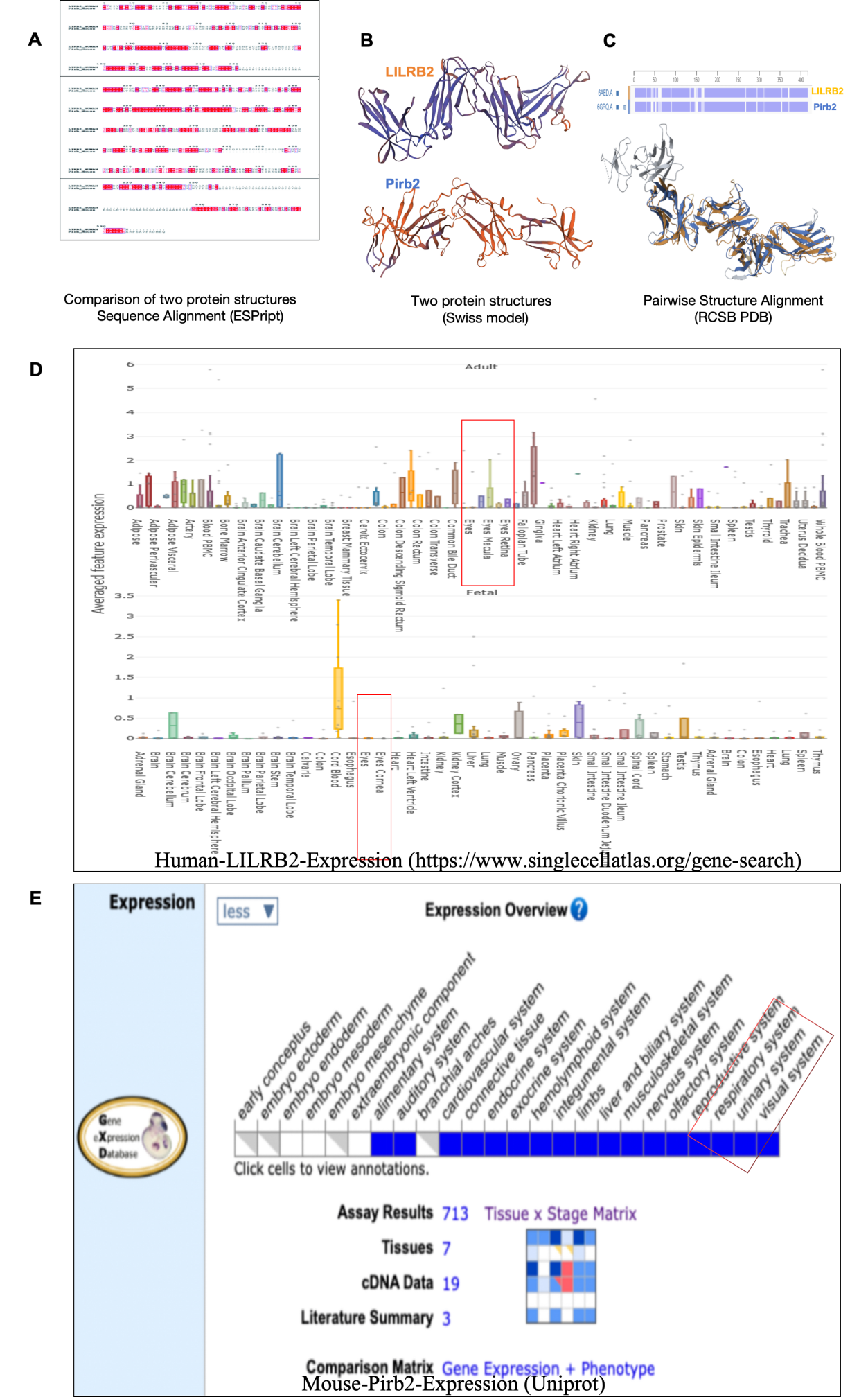


**Figure S6:** **The difference between human and mouse on the structure, expression pattern and function of LILRB2/Pirb.**

1. After using the ESPrit online tool to analyze the amino acid sequences of two proteins, it was found that the amino acid sequence similarity between human-LILRB2 protein and mouse-Pirb protein was approximately 44%. (**B**) Further comparison of the protein structures through the Swiss model and RCSB PDB online tools showed significant similarity between the two proteins. (**C**) Through the utilization of the RCSB PDB online tool for comparative analysis of the overlapping structures of two proteins, it was observed that the protein structure of yellow LILRB2 exhibited significant overlap with the protein structure of blue Pirb. (**D-E**) The expression patterns of LILRB2 and Pirb in various single-cell atlas and Unipro databases. Both databases suggest that LILRB2 and Pirb are present in the visual system. This finding underscores the need for further investigation into the potential association of these proteins with the visual system in both human and murine models.

**Table S1.** Primers used in this study.

| Primer name | Primer Sequence (5’-3’) |
| --- | --- |
| rs367070-F | TTTGTGGTGGGCTGAGGGTCTCAGC |
| rs367070-R | CACCACCACGCCCGGCTAATT |
| rs367070-S | CCGCCCAGTCAGTTTCCCTC |
| rs13345069-F | GCTTCTTGTGTCTGCCCTTC |
| rs13345069-R | GACGCTGGAGCAAAAACATT |
| rs13345069-S | TCAGCAGAGA AGCATCTCGC |
| 161-INTRON-F | TCTCGGTGAGATTTGAAGAGG |
| 161-INTRON-R | GCCCTGGAAGGAAATCAGAG |
| Deletion-F1-forth | GACTTGTAAGGGTTAAAAAGCCAA |
| Deletion-R1-forth | CATCTCGATCTGCCACTGACAC |
| Deletion-R2-forth | GACAGCAGATTCTAAAACAGTGG |
| Pirb-mRNA-F^a^ | GACTTATGCCCAGGTGAAACC |
| Pirb-mRNA-R^a^ | AGATTCGGCAGCCTGATTGTT |
| LILRA3-human-mRNA-F | AGGAGTGGGGACGTGACTT |
| LILRA3-human-mRNA-R | GGTCTGGCACGGATCTGTC |
| LILRB2-human-mRNA-F | GGCGATATGGCTGTCAGTATTAC |
| LILRB2-human-mRNA-R | GTGGGTTTTGGGTAGGCTCC |

^a^ Primer sequences obtained from literature. DOI: 10.18632/oncotarget.8853.

| **Table S2. Single-nucleotide polymorphisms (SNPs) located in the same LD and associated with the myopia-related GWAS Catalog.** | | | | | | | | | | |
| --- | --- | --- | --- | --- | --- | --- | --- | --- | --- | --- |
| SNP^a^ LD | BP (hg19) | Alleles | P | OR | Loci | Reported SNP | Gene | PubMed ID | Journal | Disease/trait |
| rs28475137 | 2:145223155 | T/C | 1.46E-04 | 1.262 | 2q22.3 | rs13382811 | *ZFHX1B* | 23933737 | Hum Mol Genet | Myopia (severe) |
| rs62169220 | 2:145225071 | G/A | 1.81E-05 | 1.294 | 2q22.3 | rs13382811 | *ZFHX1B* | 23933737 | Hum Mol Genet | Myopia (severe) |
| rs13249701 | 8:121617288 | G/A | 3.64E-03 | 0.8305 | 8q24.12 | rs6469937 | *SNTB1* | 23933737 | Hum Mol Genet | Myopia (severe) |
| rs10955977 | 8:121624489 | A/G | 4.36E-03 | 0.8339 | 8q24.12 | rs6469937 | *SNTB1* | 23933737 | Hum Mol Genet | Myopia (severe) |
| rs28543014 | 14:60985004 | T/C | 1.23E-03 | 0.8263 | 14q23.1 | rs1254319 | *SIX6* | 23396134 | Nat Genet | Refractive error |
| rs4365199 | 14:60994213 | G/T | 8.62E-04 | 0.8218 | 14q23.1 | rs1254319 | *SIX6* | 23396134 | Nat Genet | Refractive error |
| rs683922 | 15:35008676 | C/T | 4.02E-03 | 1.167 | 15q14 | rs11073058 | *GJD2* | 25823570 | Nat Commun | Axial length |
| rs670352 | 15:35009676 | A/G | 4.02E-03 | 1.167 | 15q14 | rs11073058 | *GJD2* | 25823570 | Nat Commun | Axial length |
| rs549811 | 15:35006322 | T/C | 2.16E-03 | 1.183 | 15q14 | rs524952 | *GJD2* | 23396134;  20835239 | Nat Genet | Refractive error |
| rs684374 | 15:35008592 | C/G | 1.64E-03 | 1.189 | 15q14 | rs524952 | *GJD2* | 23396134;  20835239 | Nat Genet | Refractive error |
| rs549811 | 15:35006322 | T/C | 2.16E-03 | 1.183 | 15q14 | rs634990 | *GJD2, GOLGA8B, ACTC1* | 20835239 | Nat Genet | Refractive error |
| rs684374 | 15:35008592 | C/G | 1.64E-03 | 1.189 | 15q14 | rs634990 | *GJD2, GOLGA8B, ACTC1* | 20835239 | Nat Genet | Refractive error |
| rs683922 | 15:35008676 | C/T | 4.02E-03 | 1.167 | 15q14 | rs11073060 | *GJD2* | 25233373 | PLoS One | Hyperopia |
| rs670352 | 15:35009676 | A/G | 4.02E-03 | 1.167 | 15q14 | rs11073060 | *GJD2* | 25233373 | PLoS One | Hyperopia |
| rs4778904 | 15:79418856 | A/C | 3.82E-07 | 0.7589 | 15q25.1 | rs4778879 | *RASGRF1* | 23396134 | Nat Genet | Refractive error |
| rs10775219 | 15:79443894 | G/A | 3.56E-07 | 0.7582 | 15q25.1 | rs4778879 | *RASGRF1* | 23396134 | Nat Genet | Refractive error |
| rs35602654 | 15:79417527 | A/T | 1.66E-07 | 0.7457 | 15q25.1 | rs939658 | *RASGRF1* | 20835236 | Nat Genet | Refractive error |
| rs35291410 | 15:79438660 | T/C | 7.63E-07 | 0.7568 | 15q25.1 | rs939658 | *RASGRF1* | 20835236 | Nat Genet | Refractive error |
| rs12734651 | 1:41864914 | C/A | 0.008187 | 1.153 | 3q.12.12 | rs698047 | *HIVEP3* | 21640322 | Am J Hum Genet | High myopia |
| rs6678302 | 1:41865508 | A/G | 0.007778 | 1.154 | 3q.12.12 | rs698047 | *HIVEP3* | 21640322 | Am J Hum Genet | High myopia |
| rs6680922 | 1:41865541 | T/C | 0.007537 | 1.154 | 3q.12.12 | rs698047 | *HIVEP3* | 21640322 | Am J Hum Genet | High myopia |
| rs11210535 | 1:41865682 | G/A | 0.007537 | 1.154 | 3q.12.12 | rs698047 | *HIVEP3* | 21640322 | Am J Hum Genet | High myopia |
| rs7538225 | 1:41866890 | T/C | 0.006995 | 1.156 | 3q.12.12 | rs698047 | *HIVEP3* | 21640322 | Am J Hum Genet | High myopia |
| rs698047 | 1:41867932 | C/G | 0.006487 | 1.157 | 3q.12.12 | rs698047 | *HIVEP3* | 21640322 | Am J Hum Genet | High myopia |
| rs6670896 | 1:41869122 | A/G | 0.007075 | 1.156 | 3q.12.12 | rs698047 | *HIVEP3* | 21640322 | Am J Hum Genet | High myopia |
| rs6658462 | 1:41869277 | C/A | 0.007075 | 1.156 | 3q.12.12 | rs698047 | *HIVEP3* | 21640322 | Am J Hum Genet | High myopia |
| rs12437515 | 15:34706533 | T/C | 0.008752 | 1.151 | 3q.12.12 | rs589135 | 44 kb 3' of *GJD2* | 21640322 | Am J Hum Genet | High myopia |
| rs580839 | 15:34706628 | G/A | 0.009131 | 1.151 | 3q.12.12 | rs589135 | 44 kb 3' of *GJD2* | 21640322 | Am J Hum Genet | High myopia |
| rs688220 | 15:34706674 | G/A | 0.009237 | 1.15 | 3q.12.12 | rs589135 | 44 kb 3' of *GJD2* | 21640322 | Am J Hum Genet | High myopia |
| rs2955694 | 15:34707875 | A/T | 0.006788 | 1.157 | 3q.12.12 | rs589135 | 43 kb 3' of *GJD2* | 21640322 | Am J Hum Genet | High myopia |
| rs560766 | 15:34708741 | G/A | 0.008685 | 1.152 | 3q.12.12 | rs589135 | 42 kb 3' of *GJD2* | 21640322 | Am J Hum Genet | High myopia |
| rs589135 | 15:34709241 | A/G | 0.005272 | 1.162 | 3q.12.12 | rs589135 | 42 kb 3' of *GJD2* | 21640322 | Am J Hum Genet | High myopia |
| rs524952 | 15:34713685 | T/A | 0.004856 | 1.163 | 3q.12.12 | rs589135 | 37 kb 3' of *GJD2* | 21640322 | Am J Hum Genet | High myopia |
| rs634990 | 15:34713872 | T/C | 0.004856 | 1.163 | 3q.12.12 | rs589135 | 37 kb 3' of *GJD2* | 21640322 | Am J Hum Genet | High myopia |
| rs685352 | 15:34716134 | A/G | 0.004547 | 1.165 | 3q.12.12 | rs589135 | 35 kb 3' of *GJD2* | 21640322 | Am J Hum Genet | High myopia |
| rs683922 | 15:34716475 | T/C | 0.004016 | 1.167 | 3q.12.12 | rs589135 | 35 kb 3' of *GJD2* | 21640322 | Am J Hum Genet | High myopia |
| rs670352 | 15:34717475 | G/A | 0.00402 | 1.167 | 3q.12.12 | rs589135 | 34 kb 3' of *GJD2* | 21640322 | Am J Hum Genet | High myopia |
| rs28415942 | 15:79092508 | C/T | 0.00000471 | 0.7804 | 3q.12.12 | rs28415942 | 1.7 kb 5' of *RASGRF1* | 21640322 | Am J Hum Genet | High myopia |

^a^Abbreviations: SNP, single-nucleotide polymorphism;

**Table S3. Functional annotation of the lead single-nucleotide polymorphisms in the five loci identified in this study.**

| Single-nucleotide polymorphism | Gene | HaploReg | | | | | RegulomeDB | GTEx Portal | | |
| --- | --- | --- | --- | --- | --- | --- | --- | --- | --- | --- |
|  |  | Promoter histone marks | Enhancer histone marks | DNAse | Proteins bound | Motifs changed | Score* (Prediction) | Expression Quantitative trait locus *P* value | Correlated gene | Analyzed tissue |
| rs745030 | *RASGRF1* | 5 tissues | 8 tissues | 4 tissues |  | 8 altered motifs | 2b (0.56046) | 3.80×10^−5^ | *RASGRF1* | Adipose - visceral (Omentum) |
| rs35602654 | *LINC00971* |  |  |  |  | 7 altered motifs | 3a (0.36649) | 1.80×10^−6^ | *RASGRF1* | Nerve - tibial |
| rs7247538 | *LILRB2* |  | BLD |  |  | Gm397, Mtf1 | 4 (0.60906) | - | - | - |
| rs13345069 | *LILRB2* | BLD^a^, FAT, GI^a^ | SPLN^a^ | 6 tissues |  | Hand1, RP58 | 5 (0.13454) | - | - | - |
| rs367070 | *LILRA3* |  | ESC |  |  | 4 altered motifs | 5 (0.32479) | 4.50×10^−79^ | *LILRB2* | Whole blood |

^a^BLD = blood; FAT = adipose tissue; ESC = embryonic stem cell; SPLN =spleen;

*Prediction for SNP with score: 2b, transcription factor (TF) binding + any motif + DNase footprint + DNase peak; 5, TF binding or DNase peak; 6, other; 7, no data. Tissues with the strongest significance levels are listed.

**Table S4. ALFA allele frequency of different SNPs in different populations.**

| **SNPs** | **Population** | **Sample size** | **Ref. allele** | **Alt. allele** |
| --- | --- | --- | --- | --- |
| rs13345069 | **Global** | 16332 | C=0.49418 | A=0.50582 |
|  | European | 12080 | C=0.43998 | A=0.56002 |
|  | **African** | 2816 | C=0.6697 | A=0.3303 |
|  | **African, others** | 108 | C=0.759 | A=0.241 |
|  | **African American** | 2708 | C=0.6662 | A=0.3338 |
|  | Asian | 108 | C=0.843 | A=0.157 |
|  | East Asian | 84 | C=0.87 | A=0.13 |
|  | Other Asian | 24 | C=0.75 | A=0.25 |
|  | **Latin American 1** | 146 | C=0.548 | A=0.452 |
|  | **Latin American 2** | 610 | C=0.648 | A=0.352 |
|  | **South Asian** | 94 | C=0.54 | A=0.46 |
|  | **Other** | 478 | C=0.529 | A=0.471 |
| rs367070 | **Global** | 26674 | G=0.19157 | A=0.80843 |
|  | European | 21642 | G=0.19864 | A=0.80136 |
|  | **African** | 2974 | G=0.0999 | A=0.9001 |
|  | **African Others** | 114 | G=0.175 | A=0.825 |
|  | **African American** | 2860 | G=0.0969 | A=0.9031 |
|  | Asian | 162 | G=0.481 | A=0.519 |
|  | East Asian | 134 | G=0.522 | A=0.478 |
|  | **Other Asian** | 28 | 1. G=0.29 | 1. A=0.71 |
|  | **Latin American 1** | 152 | G=0.125 | A=0.875 |
|  | **Latin American 2** | 616 | G=0.300 | A=0.700 |
|  | **South Asian** | 100 | G=0.17 | A=0.83 |

**Table S5. Gene Ontology terms significantly enriched among the candidate genes identified in this study.**

| **No.** | **Variable** | **Type** | **Ngenes** | **beta** | **beta_std** | **SE** | ***P*** | **Full_name** |
| --- | --- | --- | --- | --- | --- | --- | --- | --- |
| 1 | go_detection_of_other_organi... | set | 4 | 1.010 | 0.030 | 0.199 | 2.087×10^−7^ | go_detection_of_other_organism |
| 2 | go_pigment_granule_organizat... | set | 2 | 1.295 | 0.027 | 0.260 | 3.227×10^−7^ | go_pigment_granule_organization |
| 3 | go_mhc_protein_complex | set | 9 | 0.793 | 0.035 | 0.164 | 6.503×10^−7^ | go_mhc_protein_complex |
| 4 | go_regulation_of_t_cell_tole... | set | 4 | 0.877 | 0.026 | 0.193 | 2.859×10^−6^ | go_regulation_of_t_cell_tolerance_induction |
| 5 | gse28237_follicular_vs_early...2 | set | 55 | 0.232 | 0.025 | 0.053 | 6.657×10^−6^ | gse28237_follicular_vs_early_gc_bcell_up |
| 6 | gnf2_cd14 | set | 8 | 0.500 | 0.021 | 0.116 | 8.088×10^−6^ | gnf2_cd14 |
| 7 | gse34156_untreated_vs_24h_no...2 | set | 55 | 0.237 | 0.026 | 0.055 | 9.383×10^−6^ | gse34156_untreated_vs_24h_nod2_and_tlr1_tlr2_ligand_treated_monocyte_up |
| 8 | go_mhc_class_i_protein_compl... | set | 3 | 0.964 | 0.024 | 0.231 | 1.507×10^−5^ | go_mhc_class_i_protein_complex |
| 9 | go_beta_amyloid_binding | set | 7 | 0.662 | 0.026 | 0.159 | 1.571×10^−5^ | go_beta_amyloid_binding |
| 10 | rickman_tumor_differentiated...6 | set | 60 | 0.213 | 0.024 | 0.051 | 1.659×10^−5^ | rickman_tumor_differentiated_well_vs_poorly_up |
| 11 | suzuki_ctcfl_targets_up | set | 3 | 0.792 | 0.020 | 0.192 | 1.854×10^−5^ | suzuki_ctcfl_targets_up |
| 12 | go_mhc_class_ii_receptor_act... | set | 6 | 0.768 | 0.028 | 0.191 | 2.896×10^−5^ | go_mhc_class_ii_receptor_activity |
| 13 | go_positive_regulation_of_t_...4 | set | 9 | 0.553 | 0.024 | 0.138 | 3.099×10^−5^ | go_positive_regulation_of_t_cell_mediated_immunity |
| 14 | gse15930_stim_vs_stim_and_il...2 | set | 49 | 0.242 | 0.025 | 0.061 | 3.320×10^−5^ | gse15930_stim_vs_stim_and_il-12_24h_cd8_t_cell_up |
| 15 | molenaar_targets_of_ccnd1_an...2 | set | 18 | 0.299 | 0.019 | 0.076 | 3.879×10^−5^ | molenaar_targets_of_ccnd1_and_cdk4_up |
| 16 | go_amide_binding | set | 55 | 0.224 | 0.024 | 0.057 | 4.268×10^−5^ | go_amide_binding |
| 17 | go_maturation_of_lsu_rrna | set | 2 | 1.055 | 0.022 | 0.269 | 4.524×10^−5^ | go_maturation_of_lsu_rrna |
| 18 | gse360_ctrl_vs_l_donovani_dc...2 | set | 49 | 0.211 | 0.022 | 0.054 | 4.637×10^−5^ | gse360_ctrl_vs_l_donovani_dc_up |
| 19 | go_positive_regulation_of_t_...3 | set | 6 | 0.661 | 0.024 | 0.169 | 4.675×10^−5^ | go_positive_regulation_of_t_cell_mediated_cytotoxicity |
| 20 | go_regulation_of_tolerance_i... | set | 6 | 0.517 | 0.019 | 0.133 | 5.089×10^−5^ | go_regulation_of_tolerance_induction |
| 21 | gse25088_wt_vs_stat6_ko_macr...7 | set | 50 | 0.217 | 0.022 | 0.056 | 5.545×10^−5^ | gse25088_wt_vs_stat6_ko_macrophage_rosiglitazone_stim_up |
| 22 | gse17721_lps_vs_gardiquimod_...17 | set | 38 | 0.263 | 0.024 | 0.068 | 5.681×10^−5^ | gse17721_lps_vs_gardiquimod_8h_bmdc_dn |
| 23 | kegg_allograft_rejection | set | 11 | 0.540 | 0.026 | 0.142 | 7.046×10^−5^ | kegg_allograft_rejection |
| 24 | rickman_tumor_differentiated...4 | set | 33 | 0.247 | 0.021 | 0.065 | 7.097×10^−5^ | rickman_tumor_differentiated_well_vs_moderately_up |
| 25 | reactome_formation_of_fibrin... | set | 5 | 0.540 | 0.018 | 0.142 | 7.110×10^−5^ | reactome_formation_of_fibrin_clot_clotting_cascade |
| 26 | rodrigues_ntn1_targets_dn | set | 45 | 0.222 | 0.022 | 0.058 | 7.523×10^−5^ | rodrigues_ntn1_targets_dn |
| 27 | kegg_graft_versus_host_disea... | set | 11 | 0.527 | 0.026 | 0.140 | 8.441×10^−5^ | kegg_graft_versus_host_disease |
| 28 | uzonyi_response_to_leukotrie... | set | 12 | 0.437 | 0.022 | 0.117 | 9.109×10^−5^ | uzonyi_response_to_leukotriene_and_thrombin |
| 29 | go_blood_coagulation_intrins... | set | 4 | 0.561 | 0.016 | 0.151 | 9.894×10^−5^ | go_blood_coagulation_intrinsic_pathway |
| 30 | reactome_intrinsic_pathway | set | 40 | 0.561 | 0.016 | 0.151 | 9.894×10^−5^ | reactome_intrinsic_pathway |
| 31 | mcmurray_tp53_hras_cooperati...2 | set | 5 | 0.639 | 0.021 | 0.172 | 9.937×10^−5^ | mcmurray_tp53_hras_cooperation_response_up |

Output columns per variable in this table:

Variable: name of the gene set, gene covariate, or interaction. Names in this column are capped at 30 (by default) characters to keep the output file more readable; Type: denotes the type of variable, either SET or COVAR for normal gene sets and gene covariates provided in the input files or INTER-SS or INTER-SC, for internally created interaction terms (set by set and set by covariate, respectively);

Ngens: the number of genes in the data that are in the set (for gene sets and set-by-covariate interactions), that are in the interaction set (for set-by-set interactions), or for which non-missing values were available (for gene covariates); beta: the regression coefficient of the variable; beta_std: the semi-standardized regression coefficient, corresponding to the predicted change in Z-value given a change of one standard deviation in the predictor gene set/gene covariate (ie. BETA divided by the variable’s standard deviation); SE: the standard error of the regression coefficient; *P*: p-value for the parameter/variable; Full_Name: the full variable name; only included if the variable names exceed the maximum length for the VARIABLE column (and the abbreviate option is not set to file mode).
